# Synthesis of an Azide- and Tetrazine-Functionalized [60]Fullerene and Its Controlled Decoration with Biomolecules

**DOI:** 10.1021/acsomega.1c05955

**Published:** 2021-12-31

**Authors:** Vijay Gulumkar, Ville Tähtinen, Aliaa Ali, Jani Rahkila, Juan José Valle-Delgado, Antti Äärelä, Monika Österberg, Marjo Yliperttula, Pasi Virta

**Affiliations:** † Department of Chemistry, University of Turku, FI-20500 Turku, Finland; ‡ Instrument Centre, Faculty of Science and Engineering, Åbo Akademi University, FI-20500 Åbo, Finland; § Department of Bioproducts and Biosystems, Aalto University, FI-00076 Aalto, Finland; ∥ Division of Pharmaceutical Biosciences, Faculty of Pharmacy, University of Helsinki, FI-00014 Helsinki, Finland

## Abstract

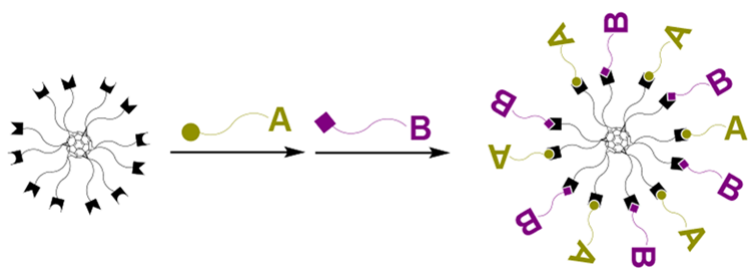

Bingel cyclopropanation between Buckminster fullerene and a heteroarmed malonate was utilized to produce a hexakis-functionalized C_60_ core, with azide and tetrazine units. This orthogonally bifunctional C_60_ scaffold can be selectively one-pot functionalized by two pericyclic click reactions, that is, inverse electron-demand Diels–Alder and azide–alkyne cycloaddition, which with appropriate ligands (monosaccharides, a peptide and oligonucleotides tested) allows one to control the assembly of heteroantennary bioconjugates.

## Introduction

Since the discovery of Buckminster fullerene ([60]fullerene, by Kroto et al.),^1^ scientists have invested significant effort by investigating potential applications of this fascinating carbon allotrope.^2^ Its synthetic availability, controlled functionalization techniques, and efficient conjugation chemistries have enabled the preparation of sophisticated C_60_ conjugates, which have had a significant impact in material and biomedical sciences.^3,4^ [60]Fullerene as such shows interesting physical and biological properties, and the spherical structure that allows radially symmetric and dense functionalization makes it an excellent scaffold to probe multivalent biomolecular interactions. For example, C_60_-based glycoballs have received marked interest as potential lectin binders.^5^ More recently, 12-armed fullerene has been used for the assembly of molecular spherical nucleic acids (SNAs),^6−8^ which is an attractive delivery and formulation option for therapeutic oligonucleotides. In most of the applications above, one type of ligand (e.g., sugars and oligonucleotides) is multiplied on an appropriately functionalized C_60_ hexakis adduct.^9−13^ However, some biomolecular applications would need a combination of different biomolecules (i.e., heteroantennary C_60_ bioconjugates). For example, a drug delivery vehicle may need a system that allows an orthogonal loading of tissue-specific and cell-penetrating ligands and the drug payloads. Appropriate heterovalency is also needed to introduce reporter groups selectively to C_60_ conjugates or to integrate them with other functionalities. A controlled mono Bingel cyclopropanation^14−16^ of [60]fullerene, followed by full-decoration with the same reaction and using two different malonates, has enabled the synthesis of 1:5-, 1:11-, and even 1:1:10-heterosubstituted C_60_ scaffolds, which have been used for an orthogonal ligation, for example, via alkyne–azide and thiol–ene click reactions.^17−25^ Recently, stereodefined C_60_ [3:3] hexa-adducts were prepared by two subsequent click reactions.^26^ For the controlled assembly, a C_60_ tris-adduct was first regioselectively prepared using a macrocyclic malonate, which was then exposed to a Bingel cyclopropanation with another malonate.

**Scheme 1:**
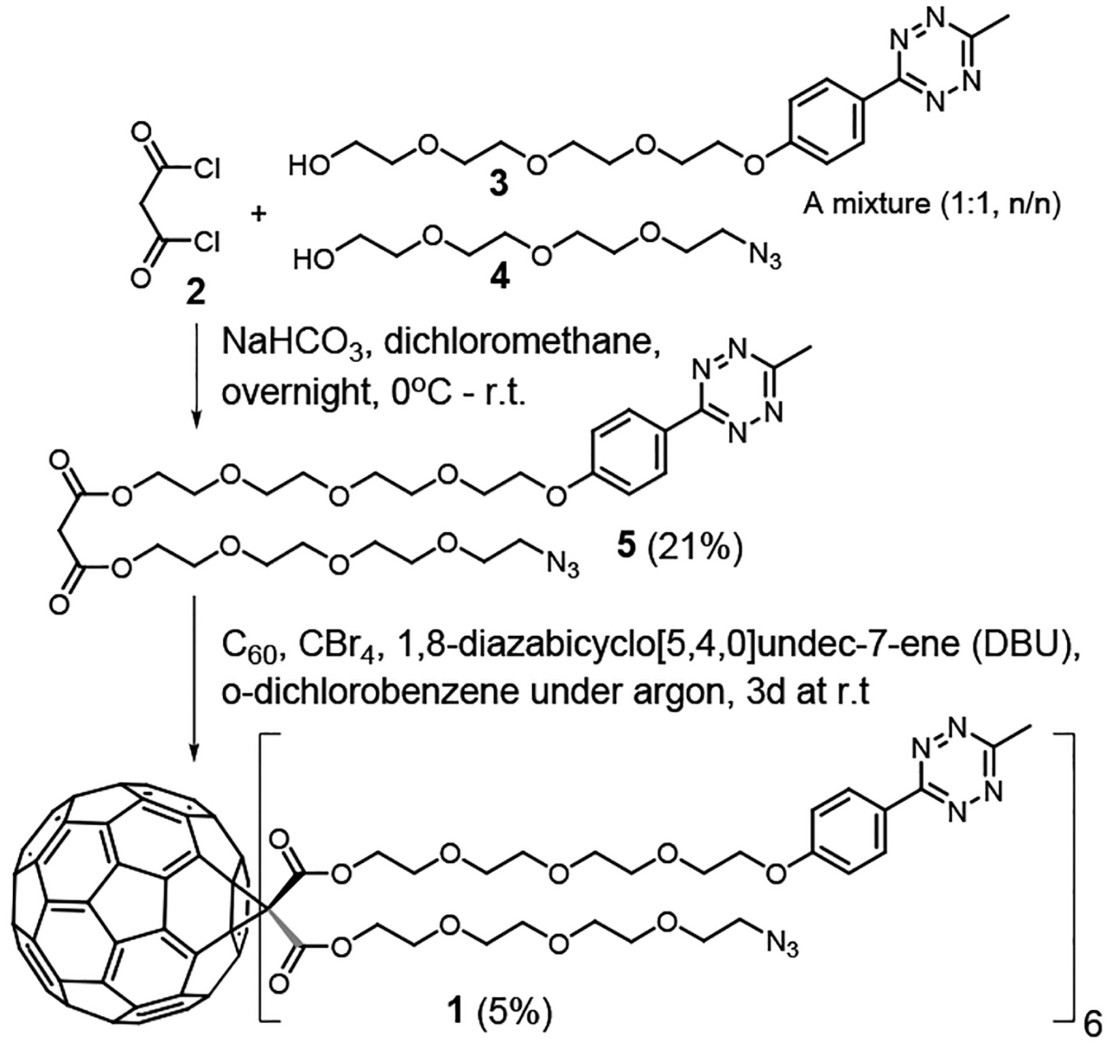
Synthesis of the Bifunctional C_60_ Core (**1**)

**Scheme 2:**
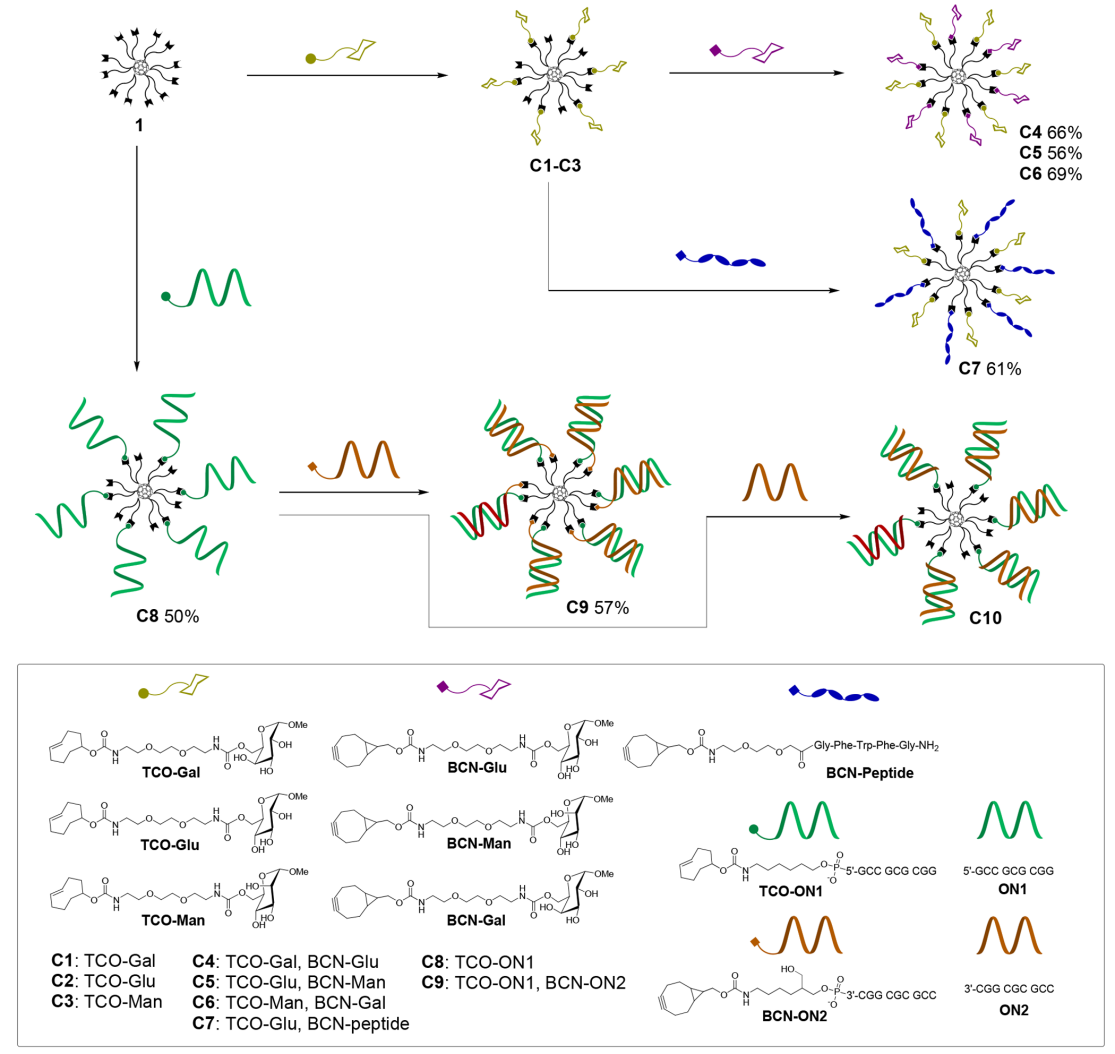
Synthesis of Hetero-Antennary Bioconjugates using the Bifunctional C_60_ Core (**1**)

The present study describes a hexakis-functionalized C_60_ core (**1**, Scheme 1), bearing azide and tetrazine units, which can be selectively one-pot functionalized by two pericyclic click reactions, that is, inverse electron-demand Diels–Alder (iEDDA) and azide–alkyne cycloaddition (SPAAC), in this order. By using this bifunctional core, heteroantennary bioconjugates (**C4**–**C9**, Scheme 2) were prepared in catalyst-free conditions using *trans-*cyclooctene (TCO)- and bicyclononyne (BCN)-modified sugars and oligonucleotides and one BCN-modified peptide.

## Results and Discussion

A Bingel cyclopropanation between [60]fullerene and heteroarm malonate **5** was used for the synthesis of **1** (Scheme 1). For the synthesis of malonate **5**, one would use Meldrum’s acid and a stepwise reaction with **3** and **4**. However, the described one step-approach was chosen, as it gave **5** in an acceptable yield (21%) with a mixture of readily availabale alcohols (Scheme S1). A Bingel cyclopropanation between C_60_ and **5** under standard conditions,^14−16^ using CBr_4_ as a bromination agent and 1,8-diazabicyclo[5.4.0]undec-7-ene (DBU) as a strong organic base in *o*-dichlorobenezene for 3 d at rt under argon, gave the hexakis-substituted C_60_ core **1**. The crude product mixture was purified first by silica gel column chromatography (in 24% yield) and then further by reversed-phase (RP) high-performance liquid chromatography (HPLC) to give homogenized **1** in 5% overall yield. The authenticity of **1** was verified by NMR spectroscopy and mass spectrometry (MS) (electrospray ionization–time-of-flight (ESI-TOF)) (Figures S5–S8). It may be worth of mentioning that, while the cyclopropanated C_60_ moiety of **1** is a well-organized structure, with pyritohedral symmetry, **1** is obtained as a stereoisomeric mixture (in fact 2^5^ = 32 steroisomers) due to the heteroarm malonate. The corresponding stereodefined structure would increase the biological and supramolecular value of these C_60_-derivatives. However, prior to a marked synthetic effort, needed to obtain the corresponding stereodefined C_60_ derivative,^26^ we wanted to evaluate first in this article how the tetrazine-azide combination on **1** works for the catalyst free assembly of heteroarm bioconjugates.

A set of TCO- and BCN-modified biomolecule ligands (cf. the tool box in Scheme 2) was used for the decoration of **1**. The ligands were prepared by a carbamate coupling from commercially available TCO and BCN precursors, an amino-modified peptide, amino-modified oligonucleotides, and *p*-nitrophenyl carbonate-modified sugars^27^ (cf. Supporting Information, Schemes S2 and S3).

**Figure 1:**
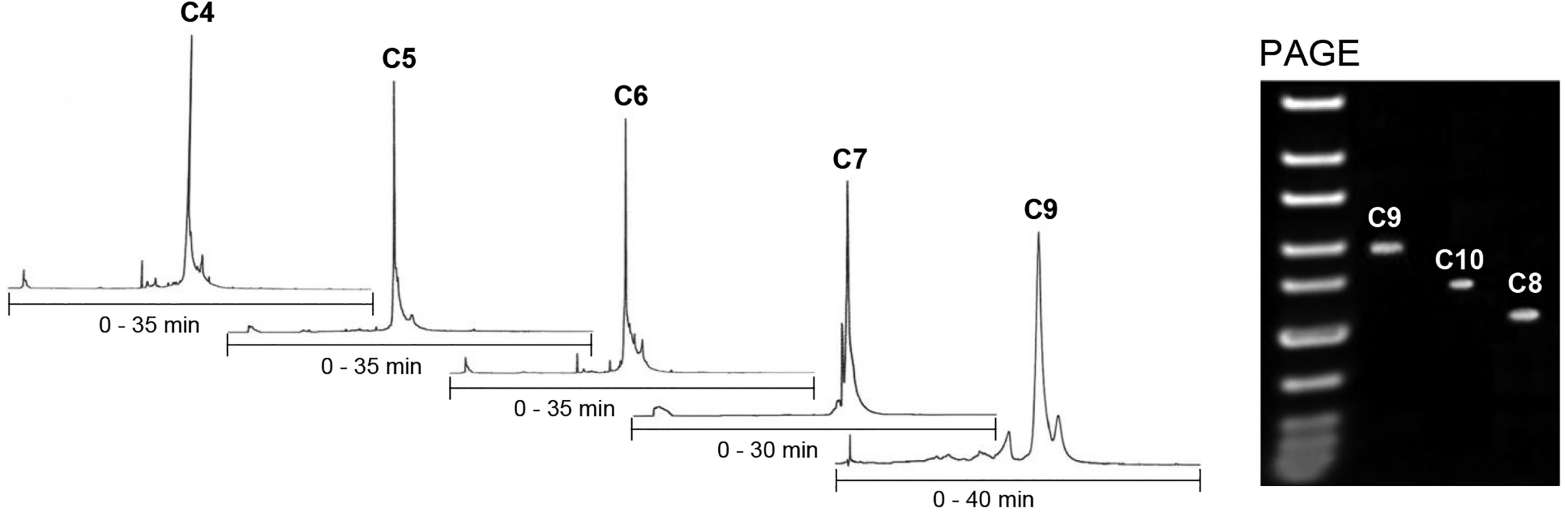
RP HPLC profiles of the crude end products **C4**–**C7** and **C9**) and PAGE electrophoregram of **C8**–**C10**. The conditions are described in the Experimental Section.

To evaluate the applicability of core **1** in the preparation of heteroarm bioconjugates, we first synthesized C_60_-glycoconjugate **C4**. **1** (0.33 μmol) was dissolved in DMSO (100 μL) and exposed to iEDDA with **TCO-Gal** (9 equiv in 100 μL of DMSO added) to yield intermediate glycoconjugate **C1**, and then **BCN-Glu** (9 equiv in 100 μL of DMSO) was added to the same reaction mixture. The completion of the successive click reactions (both overnight reactions) was verified by RP HPLC (Figure 1 and Scheme S4), and the authenticity of the intermediate conjugate **C1** and of the end product **C4** was verified by MS (ESI-TOF) (Figures S15 and S16). The isolated **C4** was characterized also by NMR spectroscopy (^1^H, COSY, HSQC, HMBC), in which the correct 1:1 ratio of the galactose and glucose units could be clearly seen (Figures S17–S20 and Table S1). Because of the small-scale synthesis, the molar quantity of the obtained **C4** was extracted from the ^1^H NMR spectra by comparing the intensity of ^1^H signals to an internal standard (a known quantity of acetonitrile used). Accordingly, **C4** was obtained in 66% isolated yield. The other heteroarm glycoconjugates **C5** and **C6** and the peptide-glycoconjugate **C7** were next assembled in a similar manner by using 0.1 μmol of **1** and adjusting the excesses of the reagents and solvent volumes accordingly. As seen in the RP HPLC profiles (Figure 1) of the crude product mixtures, the selective assembly on the C_60_ core (**1**) worked well in each case to yield the desired heteroarm conjugates **C5**–**C7** in 56, 69, and 61% isolated yields (based on the absorbance at λ = 260 nm of the products compared to that of a known concentration of **C4**, ε = 120 × 10^3^ L mol^–1^ cm^–1^). It may be worth mentioning that the idea of these small-scale trials was to further evaluate the proof of concept, in which the applicability of the scaffold **1** for the successive one-pot iEDDA and SPAAC conjugations was validated. Because of the difficulties related to the steroisomeric mixture (and the small scale), we did not invest in an effort for a complete NMR characterization, but the authenticity of the intermediate conjugates (**C2** and **C3**) and of the end products (**C5–C7**) was verified by MS (ESI-TOF) (Figures S15, S16, and S21) only.

**Figure 2:**
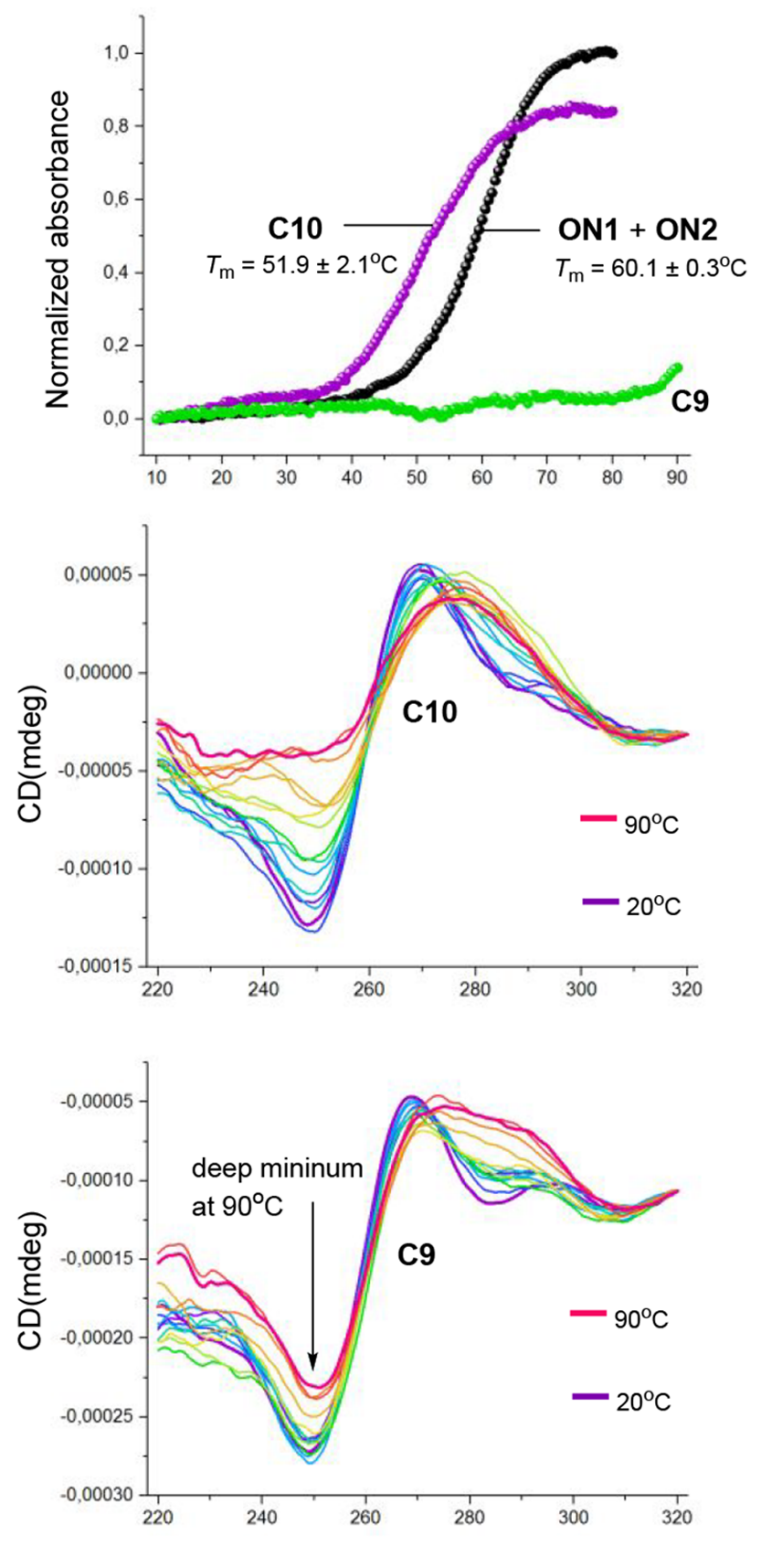
UV and CD profiles of **C8** and **C9** (ON1 + ON2 as a control). The relative hyperchromic effect is described in the UV-melting profiles. CD profiles fixed at 320 nm.

We recently communicated that multiarm C_60_-branching units (e.g., **1**) may be contaminated by structurally similar and hardly identified derivatives.^8^ Therefore, prior to the assembly of C_60_-based macromolecules, in which plausible errors may remain hidden, the homogeneity and applicability of the cores should be evaluated with small molecular ligands. After a successful synthesis of the glycoconjugates (**C4**–**C7**), we validated the applicability of **1** for the assembly of a molecularly defined SNA, in which both strands of the double helices were covalently bound to the C_60_ core (**C9**). Relatively short oligonucleotides (**TCO-ON1** and **BCN-ON2**) were used for the assembly. **1** (5 nmol) was exposed to iEDDA with **TCO-ON1** (45 nmol, 9 equiv) in DMSO (45 μL). The mixture was stirred overnight at room temperature and purified by RP HPLC (Scheme S4) to give the hexa-arm intermediate conjugate (**C8**) in 50% isolated yield (according to the UV absrobance at λ = 260 nm). The authenticity of **C8** was verified by MS (ESI-TOF) spectroscopy (Figure S22). Because of the complementarity of the **ON1** and **ON2** strands, we did not try a one-pot assembly here. An aliquot (1.0 nmol) of purified **C8** was exposed to SPAAC with **BCN-ON2** (9.0 nmol) in H_2_O (20 μL). The mixture was mixed overnight at room temperature and purified by an RP HPLC (Figure 1), which gave the homogenized SNA **C9** in 57% isolated yield (according to the UV absorbance at λ = 260 nm). As usual with SNAs, the MS-spetroscopic characterization of **C9** proved complex. An aliquot of **C9** was then introduced to Size Exclusion Chromatography equipped with a Multiple Angle Light Scattering detector (SEC-MALS), which showed a well-behaving and pure (95.7% mass fraction of the total sample mass) macromolecule with a molecular weight of 41.4 ± 1.4 kDa (expected: 41.4 kDa) (Figure S23). We also evaluated the homogeneity of **C8**, **C9**, and **C8** in the presence of complementary strand (6 equiv of **ON2**, that is, hybridization-mediated SNA **C10**) by native PolyAcrylamine Gel Electrophoresis (PAGE). As seen in the electrophoregram (Figure 1, cf. also Figure S25), each of the SNAs showed a distinct band. **C9** eluted markedly slower compared to **C10**. This is a surprising behavior, as the only difference between **C9** and **C10** is the covalent link of **ON2** to the C_60_ core. The thermal stability of the double helices on the SNAs **C9** and **C10** was next evaluated by a UV-melting profile (*T*_m_) analysis. **C10** resulted in an 8 °C decrease in the *T*_m_ value, when compared to the corresponding free duplex (**ON1** + **ON2**) (Figure 2). This decreased duplex stability is consistent with the previous findings of SNAs,^8,28−31^ caused by an electrostatic and steric repulsion between the densely packed oligonucleotides. Interestingly, **C9** showed no melting at all in the measured temperature range of 10–90 °C. This indicates very stable double helices on **C9**. One may wonder whether this is a result of a negligible hyperchromic effect, even if an unwinding of the strands occurs on **C9**. (Note: a relative hyperchromic effect is considered in the melting profiles, Figure 2). Circular dichroism (CD) measurements with **C9** and **C10** (Figure 2) were then performed to verify changes of helicity upon the temperature ramp. CD profiles of **C10** represent a typical B-type double helix. The characteristic minimum at 250 nm gradually disappears upon heating, which indicates a thermal denaturation of the double helices. Typical B-type CD profiles may be observed also on **C9**, but what makes these data fascinating is that there is no marked change between the profiles upon heating. In the profile even at 90 °C (the bold red line) a deep minimum at 250 nm can be seen, demonstrating that B-type double helices exist there. Very stable cyclic double helices are known structures,^32^ but **C9** seems to be an interesting example of dendritic nucleic acids, in which neighboring hairpin-type double helices stabilize each other via steric and electrostatic repulsion, in this manner preventing an unwinding of the strands. Polydisperse gold nanoparticle SNAs with covalently bound RNA double helices have been reported,^28^ but no similar stability phenomenom has been studied in detail.

**Figure 3:**
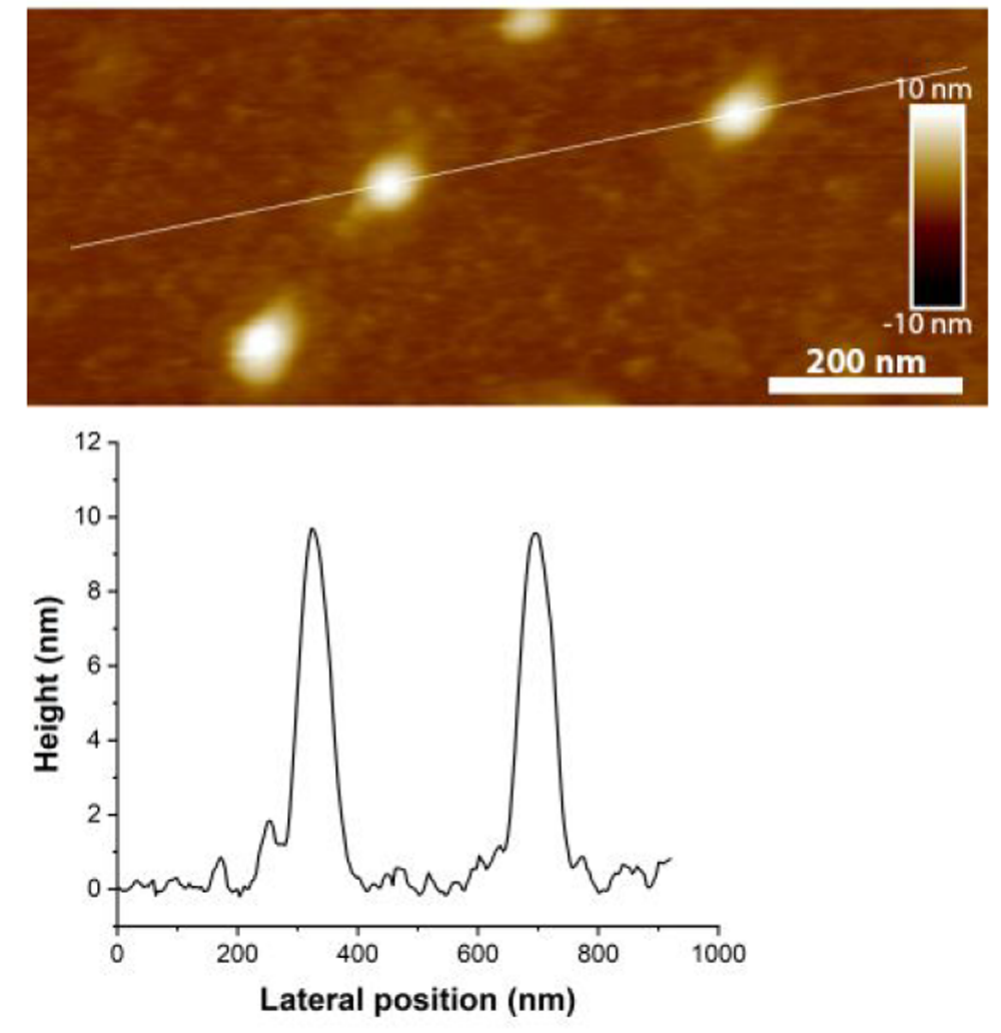
AFM height image of **C9** on PEI-coated mica in water, and a cross-section profile corresponding to the white line in the AFM image.

We also analyzed an aliquot of **C9** on a polyethylenimine (PEI)-coated mica using atomic force microscopy (AFM). Particles of ca. 10 nm height, representing the monomeric **C9** (Figure 3), were observed. We mention that the sample contained also larger but rather control-sized particles (ca. 25 nm height) that indicated aggregates of **C9** on the PEI-coated mica (Figure S24). The SNAs like **C9** may find interesting applications as well-organized constructs in medicinal and supramolecular chemistry. Further studies considering a deeper understanding of the stability requirements (the effect of the sequence and its length, hydrogen-bonding stability, Dicer-mediated cleavage^28^ of therapeutically relevant siRNAs on these SNAs, etc.) are currently under way in our laboratory.

## Conclusion

In conclusion, a bifunctional C_60_ core (**1**) has been described, which can be selectively functionalized by two subsequent pericyclic click reactions (iEDDA and SPAAC) in catalyst-free conditions. The applicability of the core in a one-pot assembly has been demonstrated for the synthesis of heteroantennary glycoballs (and one glyco-peptide C_60_-conjugate^33,34^) **C4**–**C7**, which were obtained in 56–69% isolated yields. Furthermore, a novel type SNA (**C9**) with extraordinary stable covalently bound double helices, verified by UV- and CD-melting profile experiments, has been assembled in 29% overall yield. PAGE, SEC-MALS, and AFM imaging of **C9** demonstrated a homogeneous and uniform biomacromolecule.

## Experimental Section

### General Remarks

All reactions involving air- or moisture- sensitive conditions were routinely performed and under an inert atmosphere. All reagents from commercial suppliers were used without further purification. The NMR spectra were recorded using 500 and 600 MHz instruments. The chemical shifts in the ^1^H and ^13^C NMR spectra are given in parts per million (ppm) from the residual signal of the deuterated solvents.

### RP HPLC of C_60_ Conjugates

For the analysis of the product mixture and purification of C_60_ conjugates **1**, **C1–C7**, and **C9** an analytical RP-HPLC column Phenomenex, Aeris 3.6 μm WIDEPORE XB-C8 200 Å, LC Column 150 × 4.6 mm, at a flow rate of 1.0 mL min ^–1^ and detection at 260 nm was used. Linear gradients from 40% to 100% MeCN in H_2_O over 30 min for **1**, from 0% to 100% MeCN over 30 min for **C1**–**C7**, from 0% to 100% MeCN in 50 mmol L^–1^ triethylammonium acetate over 30 min for **C8**, and from 5% to 60% MeCN in 50 mmol L^–1^ triethylammonium acetate over 40 min for **C9** were used.

### SEC-MALS

For the SEC-MALS analysis of **C9** an Agilent Technologies 1260 Infinity II HPLC system (sampler, pump, and UV/vis detector) equipped with a Wyatt Technologies miniDAWN light-scattering detector and a Wyatt Technologies Optilab refractive index detector was used. An Agilent AdvanceBio SEC 300 Å 2.7 μm 4.6 × 300 mm column, 150 mM sodium phosphate pH 7.0 as mobile phase eluting at rate of 0.2 mL min^–1^ and run time of 20 min were used. Four microliters of a sample of **C9** (1 mg mL^–1^ in Milli-Q water) was loaded onto the pre-equilibrated column. Detector signals were aligned with a bovine serum albumin (BSA) standard, which was analyzed prior to the **C9** sample. The RI and MALS signals were used for the molecular weight (MW) calculations using an average refractive index increment (d*n*/d*c*) of 0.1703 mL/g.

### PAGE Analysis of SNAs

A native 6% Tris base, boric acid, ethylenediaminetetraacetic acid (EDTA), and acrylamide (TBE) gel were used to check SNAs purity. A pre-cast gel cover 10 cm × 10 cm in size (Thermo Fisher Scientific) was fixed into a vertical electrophoresis chamber, and 10% Tris-borate-EDTA running buffer (VWR Life Science) was filled into an electrophoresis chamber. SNA samples (**C8**–**C10**, 5 μL of 0.3 μM SNAs mixed with 5 μL of 6× TriTrack DNA Loading Dye) and 5 μL of Gene Ruler Ultra Low Range DNA ladder 10–300 bp (Thermo Scientific) were loaded and electrophoresed at 150 V constant (45 mA) for ∼35 min. After the completion of the electrophoresis, the gel was removed from the electrophoresis chamber and stained by SYBRTM Gold Nucleic Acid Stain (Thermo Fisher Scientific) for 1 h and imaged under G-Box camera (Syngene).

#### Running Buffer Preparation

100 mL of (10X concentrated solution of 0.9 M Tris, 0.9 M borate, and 0.02 M EDTA, 8.3 pH in distilled, deionized water) was dissolved in 900 mL of distilled, deionized water. Sample preparations: 5 μL of (6 × TriTrack DNA Loading Dye) and 5 μL of SNAs in water (0.3 μM based on SNAs concentration). Staining solution preparation: 5 μL of SYBRTM Gold Nucleic Acid Stain was dissolved in 50 mL of running buffer.

### UV Melting Profile (*T*_m_) Experiments

The melting curves (absorbance vs temperature) were measured at 260 nm on a UV–vis spectrometer equipped with a multiple cell holder and a Peltier temperature controller. The temperature was changed at a rate of 0.5 °C min^–1^ between 10 and 80 °C (**ON1** + **ON2**-duplex and **C10**) and between 10 and 90 °C (**C9**). The measurements were performed in 10 mmol L^–1^ sodium cacodylate (pH 7.0) with 0.1 mol L^–1^ NaCl and using 1.0 μmol L^–1^**ON1** + **ON2** and 2.0 μmol L^–1^**C9** and **C10**. The *T*_m_ values were determined as the maximum of the first derivate of the melting curve. The relative increased absorbance (hyperchromicity) of the **C9** and **C10** samples was compared to that of **ON1** + **ON2**.

### CD Spectroscopic Analysis

CD spectra of **C9** and **C10** were measured on an Applied Photophysics Chirascan spectrometer. The same mixtures used for the UV melting profile analysis were used. A quartz cell of diameter 10 mm was used for the measurements. The sample temperatures were changed from 20 to 90 °C at a rate of 1 °C min^–1^.

### AFM Images

2.5 mg/mL PEI solution was deposited on cleaved mica. After 10 min of adsorption, the mica was rinsed with water and dried with nitrogen. A mixture of 10 nM **C9** in water (10 μL) was deposited on the PEI-coated mica. After 10 min of adsorption, 50 μL of water was added, and the particles were scanned in water. The AFM images were obtained using a MultiMode 8 atomic force microscope (Bruker) with a NanoScope V controller, working in tapping mode with a ScanAsyst-Fluid+ probe (Bruker).

#### 2-(2-(2-(2-(4-(6-Methyl-1,2,4,5-tetrazin-3-yl)phenoxy)ethoxy)ethoxy)ethoxy)ethanol (**3**)

4-(6-Methyl-1,2,4,5-tetrazin-3-yl)phenol (1.0 g, 5.3 mmol), 2-(2-(2-(2-hydroxyethoxy)ethoxy)-ethoxy)ethyl acetate (**6**, 1.63 g, 6.9 mmol), and triphenylphosphine (1.81 g, 6.9 mmol) were dissolved in tetrahydrofuran (25 mL) (cf. Scheme S1). The reaction mixture was cooled to 0 °C, and diisopropyl azodicarboxylate (1.35 mL, 6.8 mmol) was added dropwise over a period of 40 min. The reaction mixture was stirred for 2 h at 0 °C and for 1 h at rt. The volatiles were evaporated, and the residue was dissolved in ethyl acetate and washed with water. The organic layer was separated, dried over Na_2_SO_4_, filtered, and evaporated to dryness. The residue was purified by silica gel chromatography (*n*-hexane/ethyl acetate (EtOAc_2_), 4:6, v/v) to yield 1.59 g (74%) of 2-(2-(2-(2-(4-(6-methyl-1,2,4,5-tetrazin-3-yl)phenoxy)ethoxy)ethoxy)ethoxy)ethyl acetate (**7**) as a pink solid. ^1^H NMR (500 MHz, CDCl_3_) δ 8.53 (d, 2H, *J* = 9.0 Hz), 7.10 (d, 2H, *J* = 9.0 Hz), 4.26–4.22 (m, 4H), 3.91 (t, 2H, *J* = 4.7 Hz), 3.77–3.75 (m, 2H), 3.72–3.66 (m, 8H), 3.06 (s, 3H), 2.08 (s, 3H); ^13^C NMR (125 MHz, CDCl_3_) δ 171, 166.6, 163.8, 162.5, 129.7, 124.3, 115.3, 70.9, 70.7, 70.65, 70.6, 69.6, 69.1, 67.7, 63.6, 21, 20.9; high-resolution mass spectrometry (HRMS) (ESI-TOF) *m*/*z*: [M + Na]^+^ requires 429.1745, found 429.1745. Compound **7** (1.59 g) was dissolved in 0.1 mol L^–1^ K_2_CO_3_/MeOH solution (100 mL), and the reaction mixture was stirred for 2 h at room temperature. The completion of the acetal removal was verified by thin-layer chromatography (TLC). The reaction mixture was neutralized by an addition of 10% acetic acid (AcOH) in H_2_O and evaporated to dryness. The residue was dissolved in ethyl acetate and washed with water. The organic layer was separated, dried over Na_2_SO_4_, filtered, and evaporated to dryness. The residue was purified by silica gel chromatography (*n*-hexane/EtOAc_2_, 1:9, v/v) to yield 1 g (70%) of the product (**3**) as a pink solid. ^1^H NMR (500 MHz, CDCl_3_) δ 8.54 (d, 2H, *J* = 8.9 Hz), 7.11 (d, 2H, *J* = 9.0 Hz), 4.26 (t, 2H, *J* = 4.9 Hz), 3.92 (t, 2H, *J* = 4.7 Hz), 3.79–3.77 (m, 2H), 3.75–3.70 (m, 8H), 3.63 (t, 2H, *J* = 4.32 Hz), 3.08 (s, 3H); ^13^C NMR (125 MHz, CDCl_3_) δ 166.6, 163.8, 162.5, 129.7, 124.3, 115.3, 72.5, 70.9, 70.7, 70.6, 70.4, 69.6, 67.7, 61.8, 21.1; HRMS (ESI-TOF) *m*/*z*: [M + Na]^+^ requires 387.1639, found 387.1639.

#### Synthesis of the Heteroarm Malonate (**5**)

Tetrazine alcohol **3** (1.0 g, 2.7 mmol) and 2-(2-(2-(2-azidoethoxy)ethoxy)ethoxy)ethan-1-ol (0.6 g, 2.7 mmol) were dissolved in dry dichloromethane (DCM) (60 mL), and NaHCO_3_ (0.92 g, 4.4 equiv) was added. The reaction mixture was cooled at 0 °C, and a mixture of malonyl chloride (0.24 mL, 2.4 mmol) in dichloromethane (40 mL) was added dropwise over a period of 40 min via a dropping funnel under N_2_. The reaction mixture was allowed to warm to room temperature and stirred then overnight. The reaction was quenched by an addition of water, and the mixture was extracted with dichloromethane (twice). The organic fractions were combined, dried over Na_2_SO_4_, filtered, and evaporated to dryness. The residue was purified by silica gel chromatography (Et_2_O/acetone, 95:5, v/v) to yield 0.34 g (21%) of the product **5** as a pink oil. ^1^H NMR (600 MHz, CDCl_3_) δ 8.54 (d, 2H, *J* = 8.9 Hz), 7.11 (d, 2H, *J* = 8.9 Hz), 4.31 (t, 4H, *J* = 4.8 Hz), 4.26 (t, 2H, *J* = 4.9 Hz), 3.92 (t, 2H, *J* = 4.9 Hz), 3.78 (dd, 2H, *J* = 6.5 and 4.1 Hz), 3.74–3.67 (m, 20H), 3.46 (s, 2H), 3.40 (t, 2H, *J* = 5.0 Hz), 3.08 (s, 3H); ^13^C NMR (150 MHz, CDCl_3_) δ 166.6, 166.5, 163.8, 162.5, 129.7, 124.3, 115.3, 70.9, 70.72, 70.71, 70.9, 70.65, 70.64, 70.1, 69.6, 68.9, 67.7, 64.60, 64.58, 50.7, 41.3, 21.1; HRMS (ESI-TOF) *m*/*z*: [M+K]^+^ requires 690.2496, found 690.2498.

#### Synthesis of the Azide- and Tetrazine-Functionalized C_60_ Core (**1**)

Buckminster fullerene (37 mg, 50 μmol) was dissolved in dry and degassed (oxygen removed by bubbling with argon) *o*-dichlorobenzene (13 mL). The malonic ester (**5**, 0.33 g, 0.50 mmol), CBr_4_ (1.7 g, 5 mmol), and DBU (0.15 mL, 10 mmol) were added, and the reaction mixture was stirred for 3 d at room temperature under argon. The mixture was purified by silica gel column chromatography (DCM/MeOH 98:2, v/v) to yield 58 mg (24%) of the crude product **1**. An aliquot (25 mg) of this crude was dissolved in DMSO and introduced to RP-HPLC (Figure S5) to yield 5.5 mg (5%, overall) of the product **1** as a red sticky solid. ^1^H NMR (500 MHz, CDCl_3_): δ 8.53 (m, 2H), 7.11 (m, 2H), 4.43 (t, 2H, *J* = 4.4 Hz and m, 2H), 4.25 (b, 2H), 3.92 (b, 2H), 3.75 (b, 4H), 3.71–3.64 (m, 16H), 3.39 (t, 2H, *J* = 5.1 Hz), 3.07 (s, 3H); ^13^C NMR (125 MHz, CDCl_3_): δ 166.6, 163.8, 163.5, 162.5, 145.8, 141.0, 129.7, 124.3, 115.3, 70.9, 70.64, 70.62, 70.0, 69.6, 69.0, 68.6, 67.7, 65.86, 65.84, 50.7, 45.2, 21.1; MS (ESI-TOF): M requires 4618.6, found 4618.6 (calculated from [(M+2H)/2]^2+^.

Synthesis of BCN- and TCO-modified carbohydrates (Gal, Glu, and Man), TCO- and BCN-modified oligonucleotides (TCO-ON1 and BCN-ON2), and BCN-modified peptide. The synthetic protocols and characterization of these ligands (Scheme 2) are described in the Supporting Information (cf. Schemes S2 and S3).

#### Synthesis of Conjugate **C4**

TCO-modified methyl α-d-galactopyranoside (**TCO-Gal**, 2.9 μmol in 200 μL of DMSO) was added to a mixture of **1** (0.33 μmol in 100 μL of DMSO) in a microcentrifuge tube, and the mixture was mixed overnight at room temperature. The completion of the reaction was verified by RP-HPLC (Scheme S4), and the authenticity of the obtained intermediate product (**C1**) was verified by MS (ESI-TOF) (Figure S15). BCN-modified α-d-glucopyranoside (**BCN-Glu**, 2.9 μmol in 100 μL of DMSO) was then added. The mixture was mixed overnight at room temperature and introduced as such to an RP HPLC. The product fractions were combined and lyophilized to give homogeneous **C4**. The authenticity of the product was verified by MS (ESI-TOF) (Figure S16) and by ^1^H NMR (Figure S17), HSQC (Figures S18 and S19), and HMBC (Figure S20) spectroscopy. The assignment of the signals is shown in Table S1. ^1^H NMR was also used to determine the accurate quantity of the product, by using a known amount of acetonitrile as an internal standard and comparing the ^1^H NMR peak areas of **C4** to the methyl signal of acetonitrile. **C4** was obtained in 66% isolated yield.

#### Synthesis of Conjugates **C5–C7**

The same procedure described for **C4** was used to obtain two other C_60_-glyco conjugates **C5** and **C6** and C_60_-glyco-peptide-conjugate **C7**. 0.1 μmol of **1** was used for the assembly. The reagent excesses and solvent volumes were adjusted accordingly. The RP HPLC analysis of the individual reaction steps is described in Scheme S4. The authenticity of the intermediate products (**C2** and **C3**, aliquots purified prior MS-characterization) and of the end products (**C5**–**C7**) was verified by MS (ESI-TOF) spectroscopy (Figures S15, S16, and S21). The isolated yields were extracted from the UV absorbance of the isolated products at λ = 260 nm. (The absorbance was compared to that of a known concentration of **C4**, ε = 120 × 10^3^ L mol^–1^ cm^–1^; in the case of **C7**, the absorbance of the peptide moiety was considered.) Accordingly, **C5**, **C6**, and **C7** were isolated in 56, 69, and 61% yields, respectively.

#### Synthesis of **C8**

TCO-ON1 (40 nmol in 40 μL of DMSO) was added to a mixture of **1** (5.0 nmol) in DMSO (5 μL) in a microcentrifuge tube. The mixture was mixed overnight at room temperature. Reaction mixture was introduced to an RP-HPLC (Scheme S4), and the product fractions were lyophilized to dryness. **C8** was obtained in 50% isolated yield (based on UV absorbance at λ = 260 nm). The authenticity and homogeneity of **C8** was verified by MS (ESI-TOF) (Figure S22) and by PAGE (Figure 1).

#### Synthesis **C9**

BCN-ON2 (9.0 nmol in 10 μL of H_2_O) was added to a mixture of **C8** (1.0 nmol) in H_2_O (10 μL) in a microcentrifuge tube. The mixture was mixed overnight at room temperature and introduced to an RP HPLC (Scheme S4), and the product fractions were lyophilized to dryness. **C9** was obtained in 57% isolated yield (based on the UV absorbance at λ = 260 nm). The authenticity and homogeneity of **C9** were verified by SEC-MALS (Figure S23) and by PAGE (Figure 1).

## Supporting Information Available

Experimental and characterization details for the synthesis of **1**, **3**, **5**, BCN, and TCO-modified carbohydrates, oligonucleotides, and BCN-modified peptide and **C4**–**C9** (PDF)

## References

[ref1] Kroto H. W.; Heath J. R.; O’Brien S. C.; Curl R. F.; Smalley R. E. C_60_: Buckminsterfullerene. Nature 1985, 318, 162–163. 10.1038/318162a0.

[ref2] Cui Q.; Yang X.; Ebrahimi A.; Li J. Fullerene-biomolecule conjugates and their biomedicinal applications. Int. J. Nanomed. 2013, 9, 77–92. 10.2147/IJN.S52829. PMC3872219 24379667

[ref3] Yan W.; Seifermann S. M.; Pierrat P.; Bräse S. Synthesis of highly functionalized C_60_ fullerene derivatives and their applications in material and life sciences. Org. Biomol. Chem. 2015, 13, 25–54. 10.1039/C4OB01663G. 25329994

[ref4] Zhai W.-Q.; Jiang S.-P.; Peng R.-F.; Jin B.; Wang G.-W. Facile Access to Novel [60]Fullerenyl Diethers and [60]Fullerene-Sugar Conjugates via Annulation of Diol Moieties. Org. Lett. 2015, 17, 1862–1865. 10.1021/acs.orglett.5b00536. 25824470

[ref5] Nierengarten I.; Nierengarten J.-F. Fullerene Sugar Balls: A New Class of Biologically Active FullereneDerivatives. Chem. Asian J. 2014, 9, 1436–1444. 10.1002/asia.201400133. 24678063

[ref6] Li H.; Zhang B.; Lu X.; Tan X.; Jia F.; Xiao Y.; Cheng Z.; Li Y.; Silva D. O.; Schrekker H. S.; Zhang K.; Mirkin C. A. Molecular spherical nucleic acids. Proc. Natl. Acad. Sci. U.S.A. 2018, 115, 4340–4344. 10.1073/pnas.1801836115. 29632214 PMC5924931

[ref7] Li H.; Li Y.; Xiao Y.; Zhang B.; Cheng Z.; Shi J.; Xiong J.; Li Z.; Zhang K. Well-defined DNA–polymer miktoarm stars for enzyme-resistant nanoflares and carrier-free gene regulation. Bioconjugate Chem. 2020, 31, 530–536. 10.1021/acs.bioconjchem.0c00017. 32041403

[ref8] Gulumkar V.; Äärelä A.; Moisio O.; Rahkila J.; Tähtinen V.; Leimu L.; Korsoff N.; Korhonen H.; Poijärvi-Virta P.; Mikkola S.; et al. Controlled monofunctionalization of molecular spherical nucleic acids on a Buckminster fullerene core. Bioconjugate Chem. 2021, 32, 1130–1138. 10.1021/acs.bioconjchem.1c00187. PMC8382215 33998229

[ref9] Iehl J.; Pereira de Freitas R.; Delavaux-Nicot B.; Nierengarten J.-F. Click chemistry for the afficient preparation of functionalized [60]fullerene hesakis-addcuts. Chem. Comun. 2008, 2450–2452. 10.1039/b804393k. 18491011

[ref10] Pierrat P.; Vanderheiden S.; Muller T.; Bräse S. Functionalizatio of haxakis methanofullerene malonate crown-ethers: promising octahedarl building blocks for molecular networks. Chem. Commun. 2009, 1748–1750. 10.1039/b900367c. 19294283

[ref11] Pierrat P.; Réthore C.; Muller T.; Bräse S. Di- and dodeca-Mitsunobu reactions on C60 derivatives: Post-functionalization of fullerene mono- and hexakis-adducts. Chem. Eur. J. 2009, 15, 11458–11460. 10.1002/chem.200902141. 19821469

[ref12] Nierengarten J.-F.; Iehl J.; Oerthel V.; Holler M.; Illescas B. M.; Muñoz A.; Martin N.; Rojo J.; Sánchez-Navarro M.; Cecioni S.; et al. Fullerene sugar balls. Chem. Commun. 2010, 46, 3860–3862. 10.1039/c0cc00034e. 20414495

[ref13] Ramos-Soriano J.; Reina J. J.; Pérez-Sánchez A.; Illescas B. M.; Rojo J.; Martin N. Cyclooctyne [60]fullerene hexakis adducts: a globular scaffold for copper-free click chemistry. Chem. Commun. 2016, 52, 10544–10546. 10.1039/C6CC05484F. 27492263

[ref14] Bingel C. Cyclopropanierung von fullerenen. Chem. Ber. 1993, 126, 1957–1959. 10.1002/cber.19931260829.

[ref15] Hirsch A.; Lamparth I.; Groesser T.; Karfunkel H. R. Regiochemistry of multiple additions to the fullerene core: synthesis of a *T*_*h*_-symmetric hexakisadduct of C_60_ with bis(ethoxycarbonyl)methylene. J. Am. Chem. Soc. 1994, 116, 9385–9386. 10.1021/ja00099a088.

[ref16] Lamparth I.; Maichle-Mossmer C.; Hirsch A. Reversible template-directed activation of equatorial double bonds of the fullerene framework: regioselective direct synthesis, crustal structure, and aromatic properties of *T*_*h*_-C_66_(COOEt)_12_. Angew. Chem., Int. Ed. Engl. 1995, 34, 1607–1609. 10.1002/anie.199516071.

[ref17] Constant C.; Albert S.; Zivic N.; Baczko K.; Fensterbank H.; Allard E. Orthogonal functionalization of a fullerene building block through copper-catalyzed alkyne–azide and thiol–maleimide click reactions. Tetrahedron 2014, 70, 3023–3029. 10.1016/j.tet.2014.02.086.

[ref18] Iehl J.; Nierengarten J.-F. A click–click approach for the preparation of functionalized [5:1]-Hexaadducts of C_60_. Chem. Eur. J. 2009, 15, 7306–7309. 10.1002/chem.200901291. 19579241

[ref19] Muñoz A.; Sigwalt D.; Illescas B. M.; Luczkowiak J.; Rodriguez-Pérez L.; Nierengarten I.; Holler M.; Remy J.-S.; Buffet K.; Vincent S. P.; et al. Synthesis of giant globular multivalent glycofullerenes as potent inhibitors in a model of Ebola virus infection. Nat. Chem. 2016, 8, 50–56. 10.1038/nchem.2387. 27055288

[ref20] Trinh T. M. N.; Holler M.; Schneider J. P.; Garcia-Moreno M. I.; Garcia Fernandez J. M.; Bodlenner A.; Compain P.; Ortiz Mellet C.; Nierengarten J.-F. Construction of giant glycosidase inhibitor from iminosugar-substituted fullerene macromonomers. J. Mater. Chem. B 2017, 5, 6546–6556. 10.1039/C7TB01052D. 32264416

[ref21] Abellan Flos M.; Garcia Moreno M. I.; Ortiz Mellet C.; Garcia Fernandez J. M.; Nierengarten J.-F.; Vincent S. P. Potent glycosidase inhibition with heterovalent fullerenes: Unveiling the binding modes triggering multivalent inhibition. Chem. Eur. J. 2016, 22, 11450–11460. 10.1002/chem.201601673. 27374430

[ref22] Xu Y.; Kaur R.; Wang B.; Minameyer M. B.; Gsänger S.; Meyer B.; Drewello T.; Guldi D. M.; von Delius M. Concave-convex π-π template approach enables the synthesis of [10]cycloparaphenylene-fullerene [2]rotaxanes. J. Am. Chem. Soc. 2018, 140, 13413–13420. 10.1021/jacs.8b08244. 30234982

[ref23] Ramos-Soriano J.; Reina J. J.; Illescas B. M.; de la Cruz N.; Rodríguez-Pérez L.; Lasala F.; Rojo J.; Delgado R.; Martín N. Synthesis of highly efficient multivalent disaccharide /[60]fullerene nanoballs for emergent viruses. J. Am. Chem. Soc. 2019, 141, 15403–15412. 10.1021/jacs.9b08003. 31469952

[ref24] Ramos-Soriano J.; Reina J. J.; Illescas B. M.; Rojo J.; Martin N. Maileimide and cyclooctyne-based hexakis-adducts of fullerene: Multivalent scaffolds for copper-free click chemistry on fullerenes. J. Org. Chem. 2018, 83, 1727–1736. 10.1021/acs.joc.7b02402. 29310437

[ref25] Iehl J.; Nierengarten J.-F. Sequential copper catalyzed alkyne-azide and thiol-ene click reactions for multiple functionalization of fullerene hexaadducts. Chem. Commun. 2010, 46, 4160–4162. 10.1039/c0cc00252f. 20458384

[ref26] Meichsner E.; Schillinger F.; Trinh T. M. N.; Guerra S.; Hahn U.; Nierengarten I.; Holler M.; Nierengarten J.-F. Regioselective Synthesis of fullerene tris-adducts for the preparation of clickable fullerene [3:3]-hexa-adduct scaffolds. Eur. J. Org. 2021, 2021, 3787–3797. 10.1002/ejoc.202100572.

[ref27] Virta P.; Karskela M.; Lönnberg H. Orthogonally protected cyclo-β-tetrapeptides as solid-supported scaffolds for the synthesis of glycoclusters. J. Org. Chem. 2006, 3, 1989–1999. 10.1021/jo052348o. 16496985

[ref28] Yamankurt G.; Stawicki R. J.; Posadas D. M.; Nguyen J. Q.; Carthew R. W.; Mirkin C. A. The effector mechanism of siRNA spherical nucleic acids. Proc. Natl. Acad. Sci. U. S. A. 2020, 117, 1312–1320. 10.1073/pnas.1915907117. 31900365 PMC6983385

[ref29] Cutler I. J.; Zhang K.; Zheng D.; Auyeung E.; Prigodich E.; Mirkin C. A. Polyvalent nucleic acid nanostructures. J. Am. Chem. Soc. 2011, 133, 9254–9257. 10.1021/ja203375n. 21630678 PMC3154250

[ref30] Fong L.-K.; Wang Z.; Schatz G. C.; Luijten E.; Mirkin C. A. The role of structural enthalpy in spherical nucleic acid hybridization. J. Am. Chem. Soc. 2018, 140, 6226–6230. 10.1021/jacs.8b03459. 29762017 PMC6001361

[ref31] Randeria P. S.; Jones M. R.; Kohlstedt K. L.; Banga R. J.; Olvera de la Cruz M.; Schatz G. C.; Mirkin C. A. What controls the hybridization thermodynamics of spherical nucleic acids?. J. Am. Chem. Soc. 2015, 137, 3486–3489. 10.1021/jacs.5b00670. 25738968 PMC5490082

[ref32] El-Sagheer A. H.; Kumar R.; Findlow S.; Werner J. M.; Lane A. N.; Brown T. A very stable cyclic DNA miniduplex with just two base pairs. ChemBioChem. 2008, 9, 50–52. 10.1002/cbic.200700538. 18058775

[ref33] Pochkaeva E. I.; Podolsky N. E.; Zakusilo D. N.; Petrov A. V.; Charykov N. A.; Vlasov T. D.; Penkova A. V.; Vasina L. V.; Murin I. V.; Sharoyko V. V.; Semenov K. N. Fullerene derivatives with aminoacids, peptides and proteins: From synthesis to biomedical application. Prog. Solid. State Chem. 2020, 57, 100255 10.1016/j.progsolidstchem.2019.100255.

[ref34] Ruiz-Santaquiteria M.; Illescas B. M.; Abdelnabi R.; Boonen A.; Mills A.; Marti-Mari O.; Noppen S.; Neyts J.; Schols D.; Gago F.; San-Felix A.; Camarasa M.-J.; Martin N. Multivalent Tryptophan- and Tyrosine-Containing [60]Fullerene Hexa-Adducts as Dual HIV and Enterovirus A71 Entry Inhibitors. Chem. Eur. J. 2021, 27, 10700–10710. 10.1002/chem.202101098. 33851758 PMC8361981

