# Supplementary material for: Synthesis of an Azide- and Tetrazine-Functionalized [60]Fullerene and Its Controlled Decoration with Biomolecules
Source: ACS Omega. 2021 Dec 31;7(1):1329–36. doi: 10.1021/acsomega.1c05955 (PMC8757328; doi:10.1021/acsomega.1c05955)
Supplement: Supplementary file 1 — ao1c05955_si_001.pdf [file ao1c05955_si_001.pdf]

Supporting Information to:

Synthesis of an azide- and tetrazine-functionalized [60]fullerene and its controlled decoration with biomolecules

Vijay Gulumkar[a], Ville Tähtinen[a], Aliaa Ali[a], Jani Rahkila,[b] Juan José Valle-Delgado[c], Antti Äärelä[a], Monika Österberg[c], Marjo Yliperttula[d] and Pasi Virta[a,*]

a) Department of Chemistry, University of Turku, FI-20014 Turku, Finland, b) Instrument centre, Faculty of Science and Engineering, Åbo Akademi University, FI-20500 Åbo, Finland, c) Department of Bioproducts and Biosystems, Aalto University, FI-00076 Aalto, Finland d) Division of Pharmaceutical Biosciences, Faculty of Pharmacy, University of Helsinki, FI-00014, Helsinki, Finland

Contents

Scheme S1. Synthesis of 3 S2
Figure S1. ^{1}H NMR (500 MHz, $CDCl_3$) spectrum of **3** S2
Figure S2. ^{13}C NMR (125 MHz, $CDCl_3$) spectrum of **3.** S3

Figure S3. ^{1}H NMR (600 MHz, $CDCl_3$) spectrum of **5** S4
Figure S4. ^{13}C NMR (150 MHz, $CDCl_3$) spectrum of **5** S4

Figure S5. RP HPLC profiles of A) silica gel column-purified **1** and B) RP HPLC purified **1**. C) MS (ESI-TOF) spectrum of **1** S5
Figure S6. ^{1}H NMR (500MHz, $CDCl_3$) spectrum of **1** S5
Figure S7. ^{13}C NMR (125MHz, $CDCl_3$) spectrum of **1** S6
Figure S8. HSQC spectrum of **1** S6

General procedure for the synthesis of BCN and TCO modified carbohydrates (Gal, Glu and Man) S7

Scheme S2. Synthesis of BCN- and TCO-modified carbohydrates S7
Figure S9. ^{1}H and ^{13}C NMR (600/150 MHz, $CDCl_3$) spectra of **11** S9
Figure S10. ^{1}H and ^{13}C NMR (600/150 MHz, $CDCl_3$) spectra of **12** S10
Figure S11. ^{1}H and ^{13}C NMR (600/150 MHz, $CDCl_3$) spectra of **13**. S11
Figure S12. ^{1}H and ^{13}C NMR (500/125 MHz, $CDCl_3$) spectra of **14** S12
Figure S13. ^{1}H and ^{13}C NMR (500/125 MHz, $CDCl_3$) spectra of **15** S13
Figure S14. ^{1}H and ^{13}C NMR (500/125 MHz, $CDCl_3$) spectra of **16** S14

Synthesis of TCO- and BCN-modified oligonucleotides (TCO-ON1 and BCN-ON2) and BCN-modified peptide S15

Scheme S3. Synthesis of BCN-peptide, TCO-ON1 and BCN-ON2 S16

Scheme S4. RP HPLC analyses of the conjugates S16
Figure S15. MS(ESI-TOF) spectra of C_{60}-glyco conjugates **C1-C3** S17
Figure S16. MS(ESI-TOF) spectra of C_{60}-glyco conjugates **C4-C6** S18
Figure S17. ^{1}H NMR (600MHz, d_6-DMSO) of **C4** S19
Figure S18. HSQC spectrum of **C4** S20
Figure S19. A band selective HSQC spectrum (SHSQC) of **C4** S21
Figure S20. HMBC spectrum of **C4** S22
Table S1. Assignments of 1H and 13C NMR shifts of **C4** S23
Figure S21. MS(ESI-TOF) spectrum of C_{60}-glyco/peptide conjugate **C7** S24
Figure S22. MS(ESI-TOF) spectrum of C_{60}-oligonucleotide conjugate **C8** S24
Figure S23. SEC-MALS analysis of **C9** S24
Figure S24. AFM images of C9 on polyethylene imine (PEI) coated mica S25
Figure S23. PAGE electrophoregram of C8, C9, C10 and ON2. S25
References S25

Scheme S1. Synthesis of 3. i) 4-(6-methyl-1,2,4,5-tetrazin-3-yl)phenol, DIAD, Ph_3P, THF, 2h, 0ºC – r.t., ii) K2C=3, methanol, 2h at r.t.

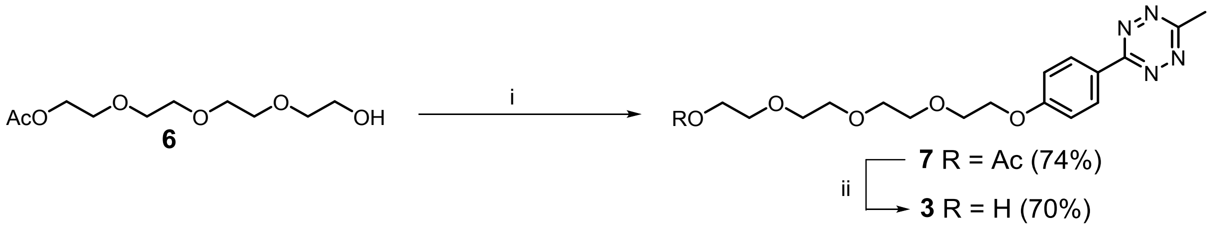

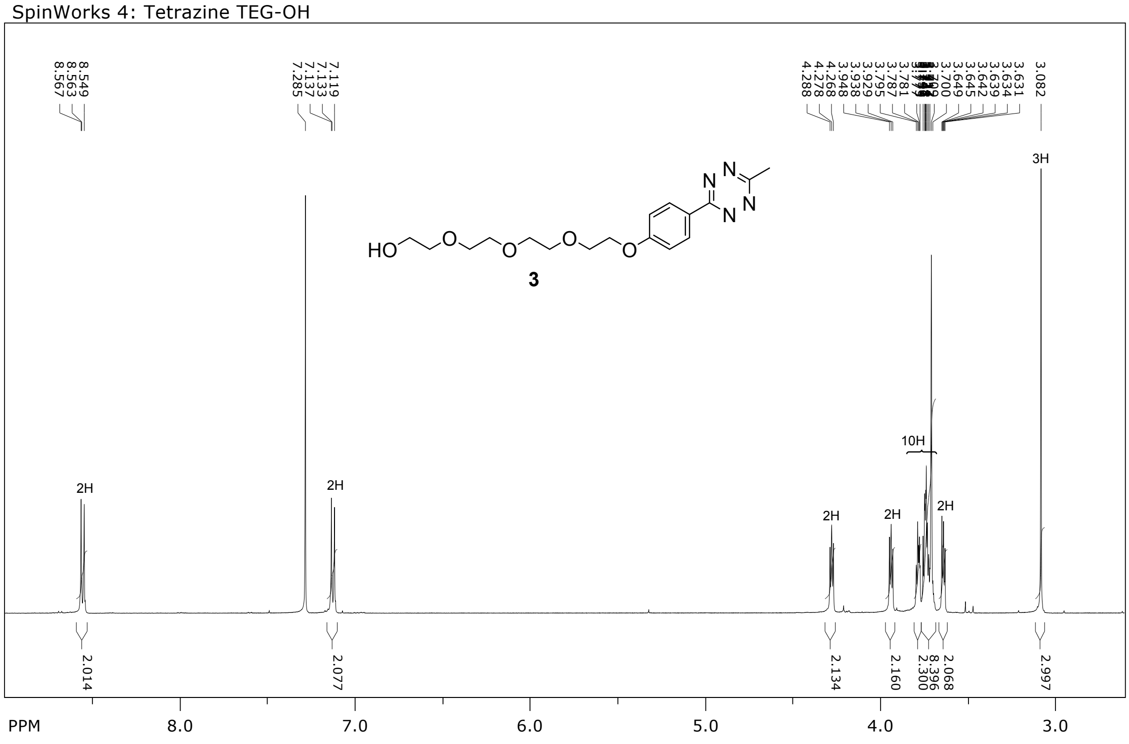

Figure S1. ^{1}H NMR (500 MHz, $CDCl_3$) spectrum of **3.**

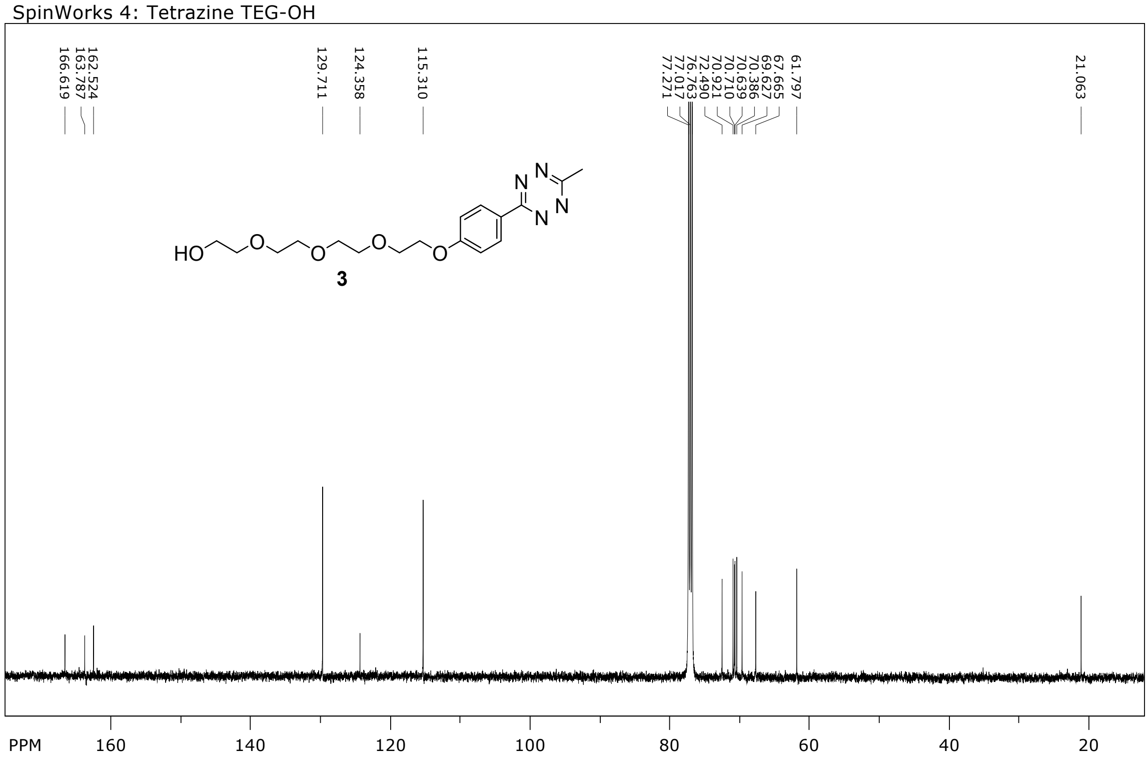

Figure S2. ^{13}C NMR (125 MHz, $CDCl_3$) spectrum of **3.**

SpinWorks 4: Azide and tetrazine modified linker

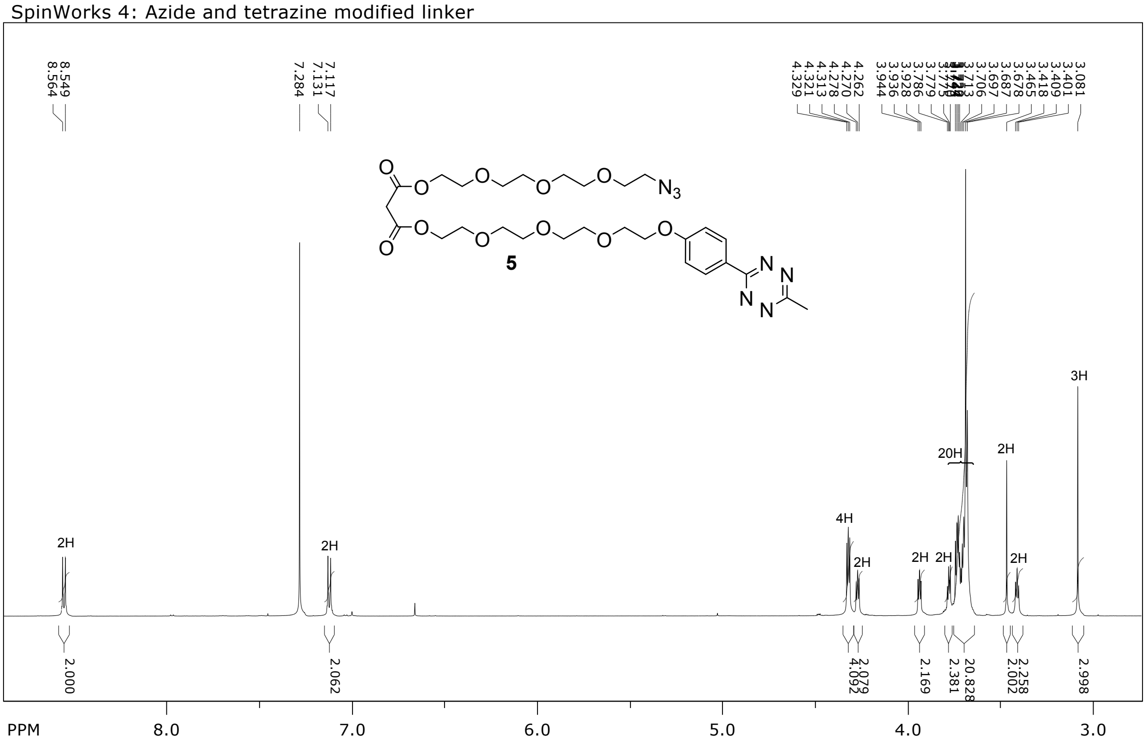

Figure S3. ^{1}H NMR (600 MHz, $CDCl_3$) spectrum of **5**

SpinWorks 4: Azide and tetrazine modified linker

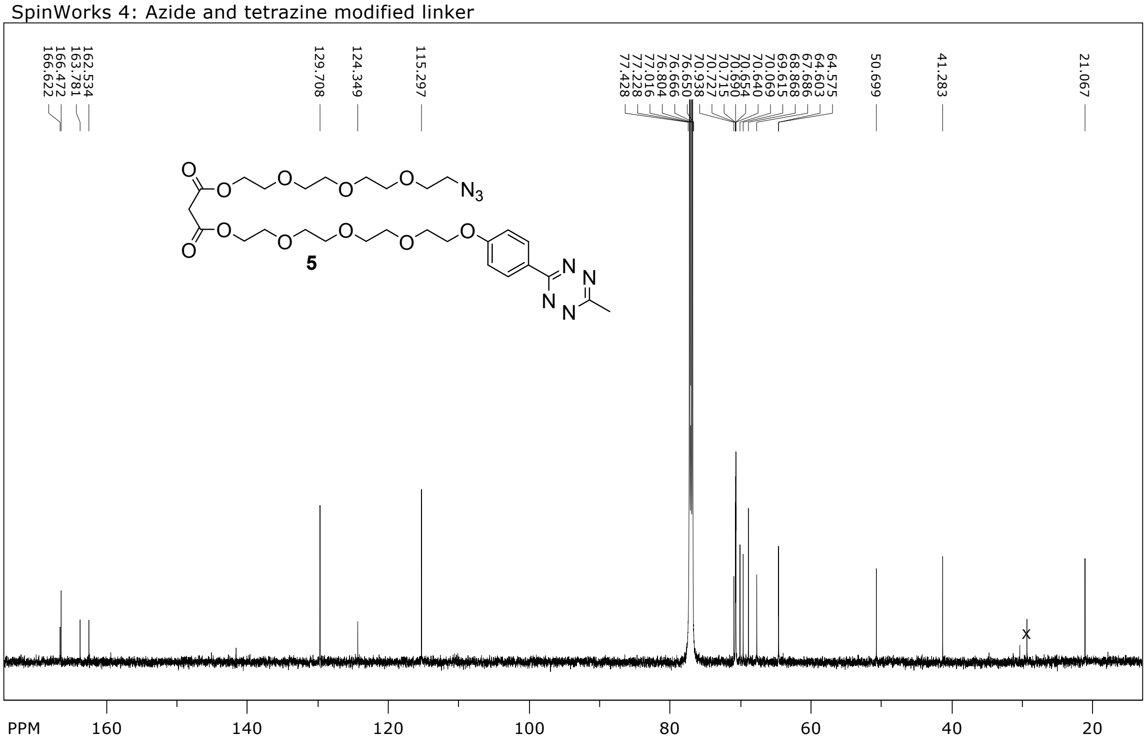

Figure S4. ^{13}C NMR (150 MHz, $CDCl_3$) spectrum of **5**

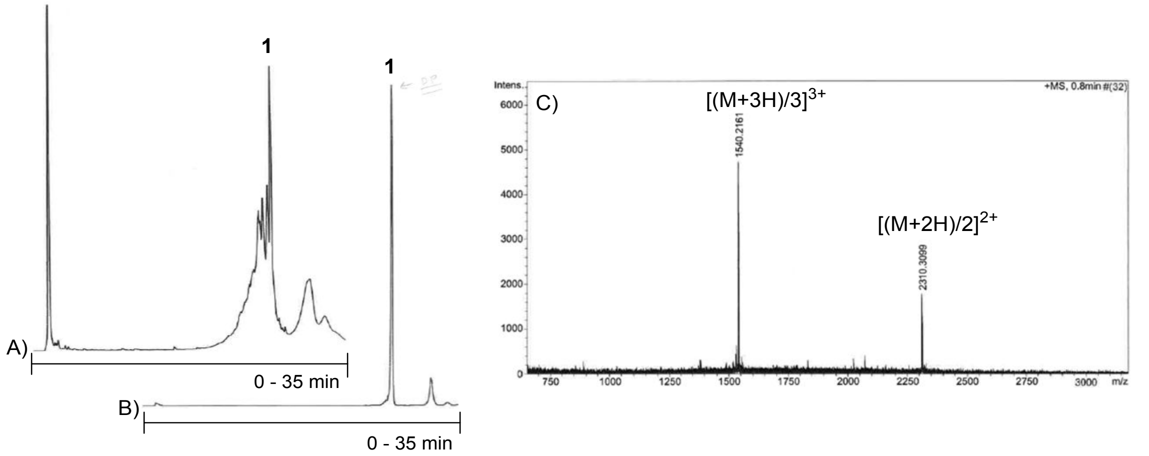

Figure S5. RP HPLC profiles of A) silica gel column-purified **1** and B) RP HPLC purified **1**. C) MS (ESI-TOF) spectrum of **1**. (RP HPLC conditions: see general procedures)

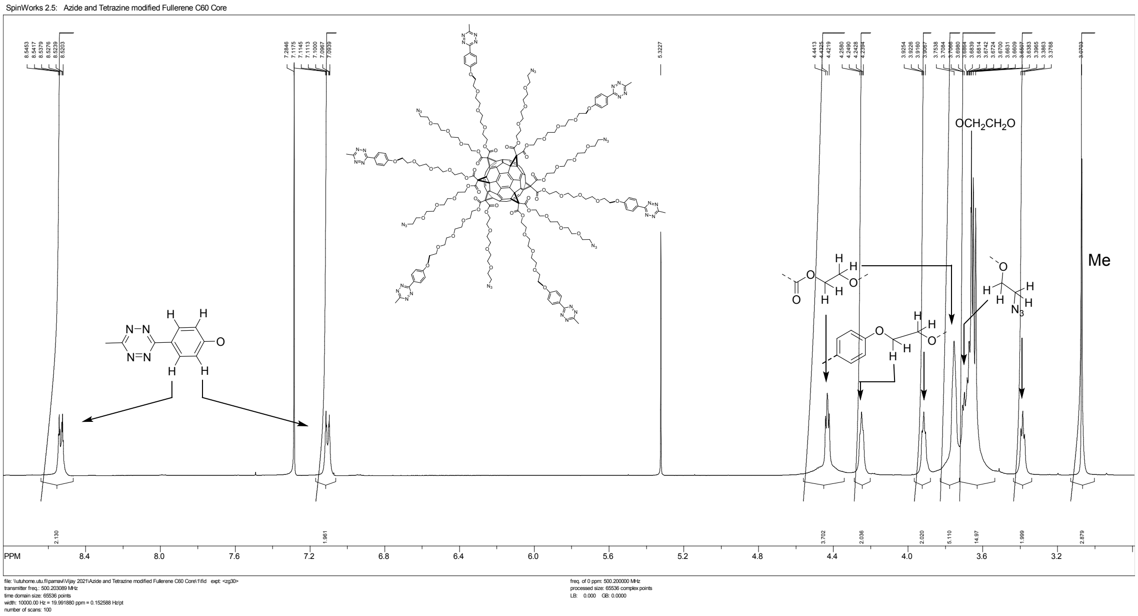

Figure S6. ^{1}H NMR (500MHz, $CDCl_3$) spectrum of **1.**

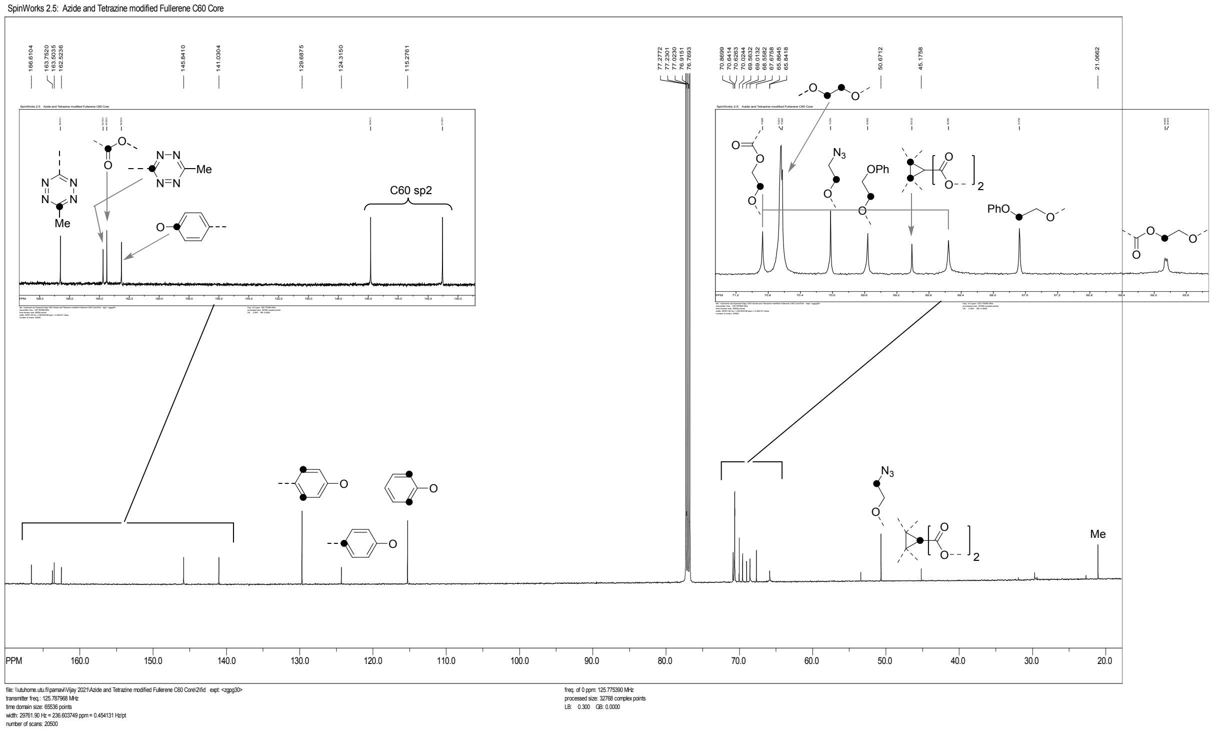

Figure S7. ^{13}C NMR (125MHz, $CDCl_3$) spectrum of **1**

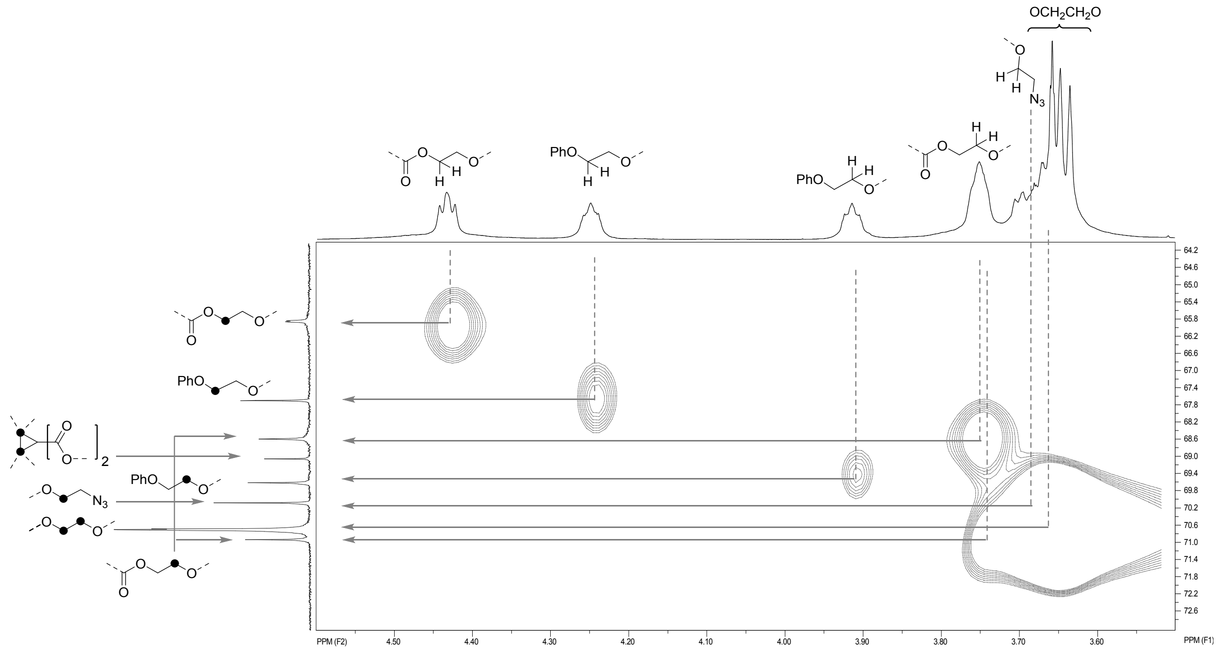

Figure S8. HSQC spectrum of **1**

Scheme S2. Synthesis of BCN- and TCO-modified carbohydrates. Reagents and conditions: i) HOBt, DIEA, 1,4-dioxane, overnight, r.t.; ii) 7N ammonia in MeOH, 55 °C.

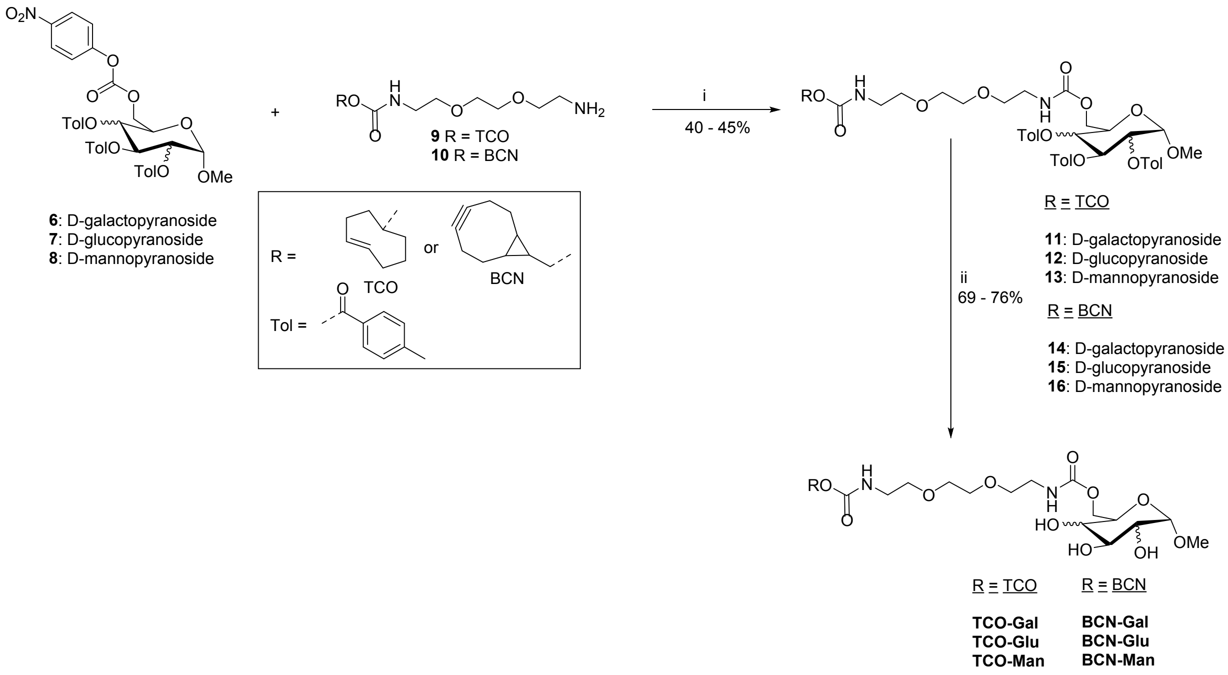

General procedure for Synthesis of BCN and TCO modified carbohydrates (Gal, Glu and Man). p-nitrophenylcarbonate-activated methyl α-D-galacto, D-gluco or D-manno pyranoside (**6**-**8**, 59.3 mg, 0.082 mmol, prepared as previously described[2]), HOBt (12.7 mg, 0.082 mmol) and DIEA (23 µL, 0.13 mmol) were dissolved in 1,4-dioxane (1 mL) and the TCO- amine (**9**, 20 mg, 0.066 mmol) or BCN-amine (**10**, 21.5 mg, 0.066 mmol) was added. The mixture was stirred overnight at room temperature and evaporated to dryness. The residue was purified by silica gel chromatography (n-Hexane/$EtOAc_2$, 4:6, v/v) to yield 23-26 mg (40-45%) of the toluoyl protected sugar products (**11** – **16**) as colorless oil.

11: ^{1}H NMR (600 MHz, $CDCl_3$) δ 7.97 (d, 2H, *J* = 8.1 Hz), 7.87 (d, 2H, *J* = 8.2 Hz), 7.68 (d, 2H, *J* = 8.2 Hz), 7.29 (d, 2H, *J* = 8.1 Hz), 7.17 (d, 2H, *J* = 8.1 Hz), 7.05 (d, 2H, *J* = 8.1 Hz), 5.92 – 5.91 (m, 2H), 5.62 (dd, 1H, *J* = 10.4 & 3.5 Hz), 5.58 – 5.48 (m, 2H), 5.33 (b, 1H), 5.30 (d, 1H, *J* = 3.5 Hz), 5.19 (b, 1H), 4.45 (dd, 1H, *J* = 6.4 Hz, both), 4.35 (1H, b), 4.33 (dd, 1H, *J* = 11.2 & 6.7 Hz), 4.18 (dd, 1H, *J* = 11.1 & 6.4 Hz), 3.63 – 3.58 (m, 4H), 3.57 – 3,52 (m, 4H), 3.48 (s, 3H), 3.39 – 3.32 (m, 4H), 2.45 (s, 3H) 2.39 – 2.33 (m, 2H), 2.36 (s, 3H), 2.31 (s, 3H), 2.02 (m, 1H), 1.97 – 1.90 (m, 2H), 1.80 – 1.71 (m, 2H), 1.54 (m, 1H); ^{13}C NMR (150 MHz, $CDCl_3$) δ 166.1, 165.6, 156.3, 155.8, 144.2, 144.1, 143.8, 135.0, 133.0, 130.0, 129.9, 129.7, 129.3, 129.1, 128.9, 126.59, 126.55, 126.52, 97.7, 80.5, 70.3, 70.24, 70.19, 70.0, 69.2, 68.9, 68.2, 66.9, 62.5, 55.7, 41.1, 40.9, 40.7, 38.7, 34.3, 32.5, 31.0, 21.7, 21.65, 21.59; HRMS (ESI-TOF) m/z: $[M+Na]^+$ requires 897.3780, found 897.3779.

12: ^{1}H NMR (600 MHz, $CDCl_3$) δ 7.88 (d, 2H, *J* = 8.1 Hz), 7.84 (d, 2H, *J* = 8.0 Hz), 7.77 (d, 2H, *J* = 8.0 Hz), 7.19 – 7.18 (m, 4H), 7.10 (d, 2H, *J* = 7.9 Hz), 6.12 (dd, 1H, *J* = 10.0 & 9.8 Hz), 5.62 – 5.49 (m, 3H), 5.32 (b, 1H), 5.25 – 5.22 (m, 2H), 5.10 (b, 1H), 4.43 – 4.33 (m, 2H), 4.30 – 4.22 (m, 2H), 3.65 -3.60 (m, 4H), 3.60 – 3.53 (m, 4H), 3.48 (s, 3H), 3.42 -3.35 (m, 4H), 2.37 (s, 6H), 2.40 – 2.32 (m, 2H), 2.31 (s, 3H), 2.03 (m, 1H), 1.98 – 1.90 (m, 2H), 1.81 – 1.70 (m, 2H), 1.35 (m, 1H); ^{13}C NMR (150 MHz, $CDCl_3$) δ 165.9, 165.8, 165.3, 156.3, 156.0, 144.1, 143.7, 135.0, 133.0, 130.0, 129.9, 129.7, 129.1, 129.0, 126.6, 126.34, 126.28, 97.1, 80.6, 71.9, 70.31, 70.27, 70.2, 70.1, 69.0, 67.9, 62.9, 55.6, 41.2, 40.9, 40.7, 38.7, 34.3, 32.5, 31.6, 31.0, 21.7, 21.6; HRMS (ESI-TOF) m/z: $[M+Na]^+$ requires 897.3780, found 897.3778.

13: ^{1}H NMR (600 MHz, $CDCl_3$) δ 7.99 (d, 2H, *J* = 8.1 Hz), 7.86 (d, 2H, *J* = 8.2 Hz), 7.73 (d, 2H, *J* = 8.2 Hz), 7.30 (d, 2H, *J* = 7.9 Hz), 7.18 (d, 2H, *J* = 8.1 Hz), 7.07 (d, 2H, *J* = 8.1 Hz), 5.84-5.83 (m, 2H), 5.63 (b, 1H), 5.59-5.48 (m, 2H), 5.27 (b, 1H), 5.14 (b, 1H), 4.99 (b, 1H), 4.40 (m, 1H), 4.34 (m, 1H), 4.32-4.27 (m, 2H),

3.59 (m, 4H), 3.54-3.52 (m, 8H), 3.38-3.35 (m, 4H), 2.45 (s, 3H), 2.37 (s, 3H), 2.38 – 2.32 (m, 2H), 2.31 (s, 3H), 2.01 (m, 1H), 1.96-1.90 (m, 2H), 1.79-1.70 (m, 2H), 1.54 (m, 1H); ^{13}C NMR (150 MHz, $CDCl_3$) δ 165.60, 165.56, 165.5, 156.3, 156.0, 144.25, 144.15, 143.8, 134.9, 133.0, 129.9, 129.85, 129.78, 129.3, 129.1, 129.0, 126.7, 126.45, 126.37, 98.6, 80.6, 70.3, 70.24, 70.21, 70.1, 68.9, 67.0, 63.7, 55.4, 41.1, 40.9, 40.7, 38.7, 34.3, 32.5, 31.0, 21.8, 21.7, 21.6; HRMS (ESI-TOF) m/z: $[M+Na]^+$ requires 897.3780, found 897.3782.

14: ^{1}H NMR (500 MHz, $CDCl_3$) δ 7.97 (d, 2H, *J* = 8.0 Hz), 7.87 (d, 2H, *J* = 8.1 Hz), 7.68 (d, 2H, *J* = 8.1 Hz), 7.29 (d, 2H, *J* = 6.9 Hz), 7.18 (d, 2H, *J* = 8.0 Hz), 7.05 (d, 2H, *J* = 8.0 Hz), 5.91 (b, 2H), 5.62 (dd, 1H, *J* = 10.8 & 3.4 Hz), 5.33 (b, 1H), 5.30 (d, 1H, *J* = 3.5 Hz), 4.45 (dd, 1H, *J* = 6.4 & 6.3 Hz), 4.33 (dd, 1H, *J* = 11.1 & 6.6 Hz), 4.19-4.15 (m, 3H), 3.62 (b, 4H), 3.58-3.54 (m, 4H), 3.48 (s, 3H), 3.39-3.36 (m, 4H), 2.46 (s, 3H), 2.37 (s, 3H), 2.31 (s, 3H), 2.33 – 2.19 (m, 6H), 1.59 (m, 2H), 1.37 (m, 1H), 0.95 (m, 2H); ^{13}C NMR (125 MHz, $CDCl_3$) δ 166.1, 165.6, 156.8, 155.8, 144.2, 144.1, 143.8, 130.0, 129.9, 129.7, 129.3, 129.1, 128.9, 126.60, 126.55, 126.52, 98.8, 97.7, 70.33, 70.29, 70.2, 70.0, 69.2, 68.9, 68.3, 66.9, 62.7, 62.4, 55.7, 40.9, 40.8, 29.1, 21.7, 21.65, 21.59, 21.4, 20.1, 17.8; HRMS (ESI-TOF) m/z: $[M+Na]^+$ requires 921.3780, found 921.3776.

15: ^{1}H NMR (500 MHz, $CDCl_3$) δ 7.86 (d, 2H, *J* = 8.1 Hz), 7.83 (d, 2H, *J* = 8.1 Hz), 7.75 (d, 2H, *J* = 8.1 Hz), 7.19-7.17 (m, 4H), 7.08 (d, 2H, *J* = 8.1 Hz), 6.11 (dd, 1H, *J* = 9.8Hz, both), 5.35 (b, 1H), 5.56 (dd, 1H, *J* = 9.8 Hz, both), 5.24-5.21 (m, 2H), 4.37 (d, 1H, *J* = 9.9 Hz), 4.28-4.22 (m, 2H), 4.17-4.15 (m, 2H), 3.64 (m, 4H), 3.61 – 3.56 (m, 4H), 3.47 (s, 3H), 3.41-3.38 (m, 4H), 2.36 (s, 6H), 2.31 (s, 3H), 2.30-2.19 (m, 6H), 1.58 (m, 2H), 1.38 (m, 1H), 0.94 (m, 2H); ^{13}C NMR (125 MHz, $CDCl_3$) δ 165.9, 165.7, 165.3, 156.8, 156.0, 144.1, 143.7, 130.0, 129.9, 129.7, 129.1, 129.0, 126.6, 126.33, 126.28, 98.8, 97.1, 71.9, 70.3, 70.2, 70.1, 69.0, 67.9, 62.9, 62.7, 55.6, 40.9, 40.8, 29.0, 21.6, 21.5, 21.4, 20.1, 17.8; HRMS (ESI-TOF) m/z: $[M+Na]^+$ requires 921.3780, found 921.3774.

16: ^{1}H NMR (600 MHz, $CDCl_3$) δ 7.96 (d, 2H, *J* = 8.0 Hz), 7.83 (d, 2H, *J* = 8.2 Hz), 7.69 (d, 2H, *J* = 8.2 Hz), 7.27 (d, 2H, *J* = 7.9 Hz), 7.15 (d, 2H, *J* = 8.0 Hz), 7.04 (d, 2H, *J* = 8.1 Hz), 5.81 (m, 2H), 5.61 (b, 1H), 5.23 (b, 2H), 4.97 (d, 1H, *J* = 0.95 Hz), 4.37 (d, 1H, *J* = 7.7 Hz), 4.28-4.26 (m, 2H), 4.17 – 4.10 (m, 2H), 3.58 (b, 4H), 3.54-3.5 (m, 4H), 3.51 (s, 3H), 3.36-3.35 (m, 4H), 2.43 (s, 3H), 2.35 (s, 3H), 2.28-2.17 (m, 6H), 1.56 (m, 2H), 1.35 (m, 1H), 0.92 (m, 2H); ^{13}C NMR (150 MHz, $CDCl_3$) δ 165.60, 165.56, 165.6, 156.8, 156.0, 144.25, 144.16, 143.8, 129.9, 129.85, 129.77, 129.3, 129.1, 129.0, 126.7, 126.44, 126.36, 98.8, 98.6, 70.4, 70.3, 70.2, 70.0, 69.8, 68.9, 67.0, 63.7, 62.7, 55.4, 40.9, 40.8, 29.0, 21.77, 21.66, 21.6, 21.4, 20.1, 17.8; HRMS (ESI-TOF) m/z: $[M+Na]^+$ requires 921.3780, found 921.3771.

20 mg samples of the toluoyl protected sugars (**11** – **16**) were dissolved in 7N ammonia in MeOH (2 mL) and the mixtures were stirred overnight at 55 °C. The complete toluoyl removal was verified by TLC, and the mixtures were evaporated to dryness. The residues were purified by filtration through a short silica gel colum (DCM/MeOH, 9:1, v/v) to yield (69-76%) of unprotected sugars **TCO-Gal**, **TCO-Glu**, **TCO-Man**, **BCN-Gal**, **BCN-Glu** and **BCN-Man** as colorless oil. The authenticity of the products was verified by HRMS (ESI-TOF). For TCO-modified sugars $[M+Na]^+$ requires 543.2524, found 543.2524 (**TCO-Gal**), 543.2525 (**TCO-Glu**), 543.2524 (**TCO-Man**), For BCN-modified sugars m/z: $[M+Na]^+$ requires 567.2524, found 567.2526 (**BCN-Gal**), 567.2523 (**BCN-Glu**) and 567.2509 (**BCN-Man**).

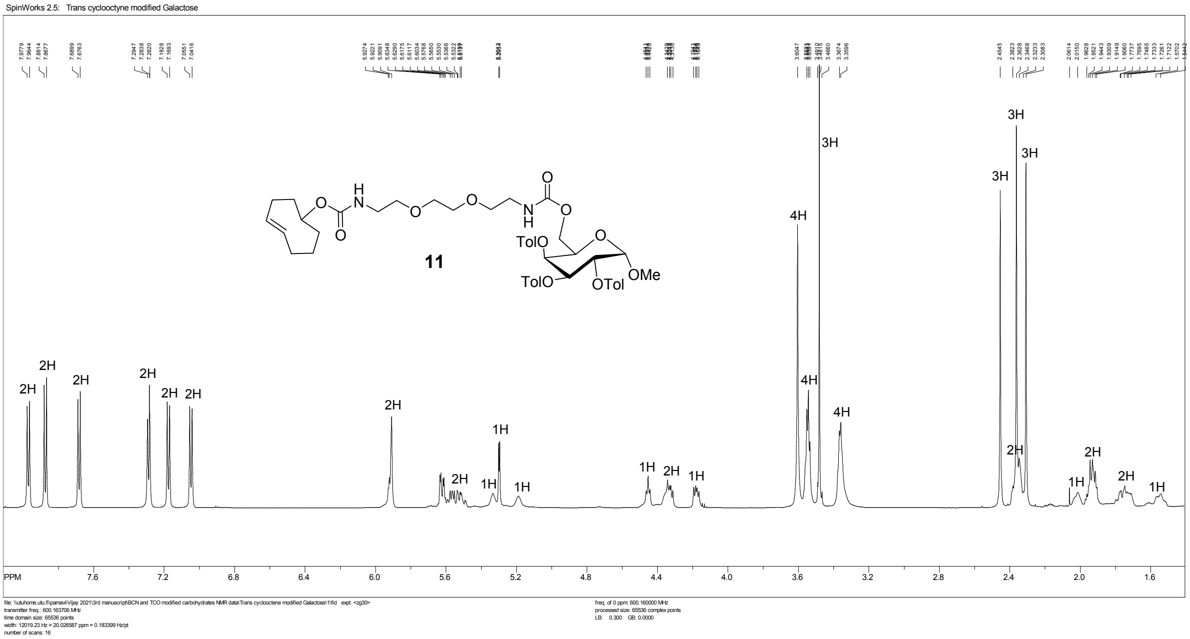

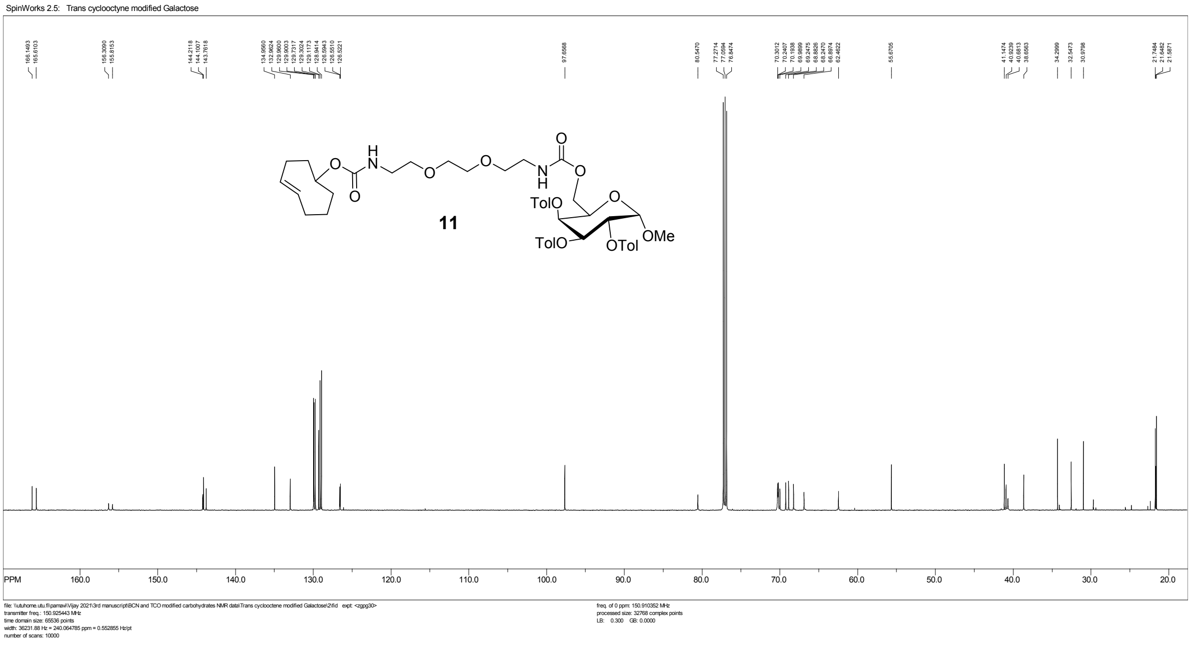

Figure S9. ^{1}H and ^{13}C NMR (600/150 MHz, $CDCl_3$) spectra of **11**.

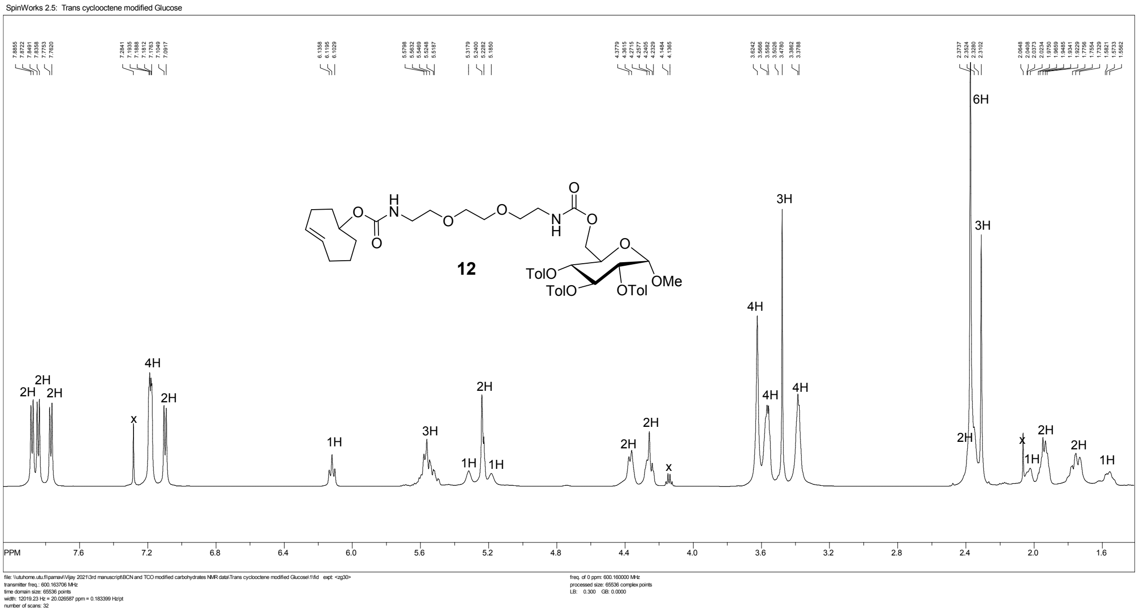

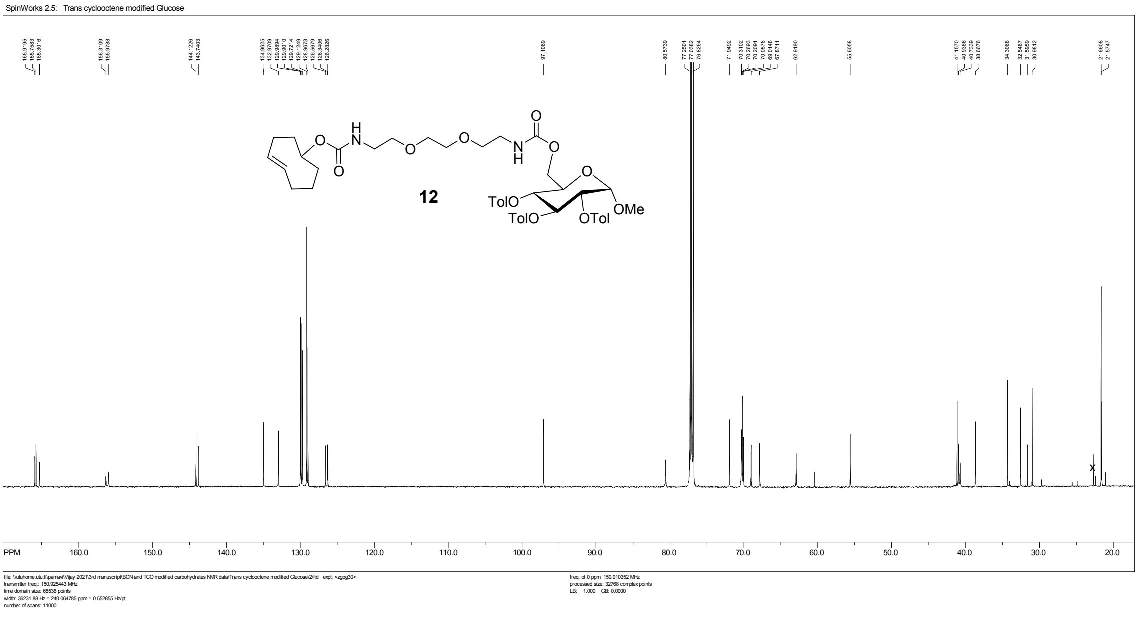

Figure S10. ^1H and ^{13}C NMR (600/150 MHz, $CDCl_3$) spectra of **12**.

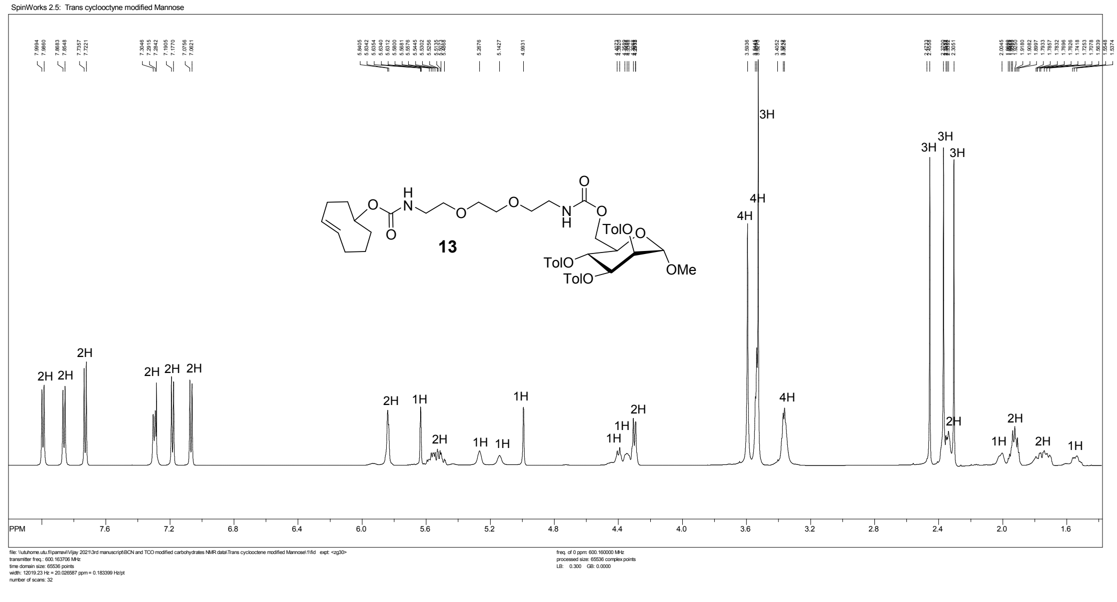

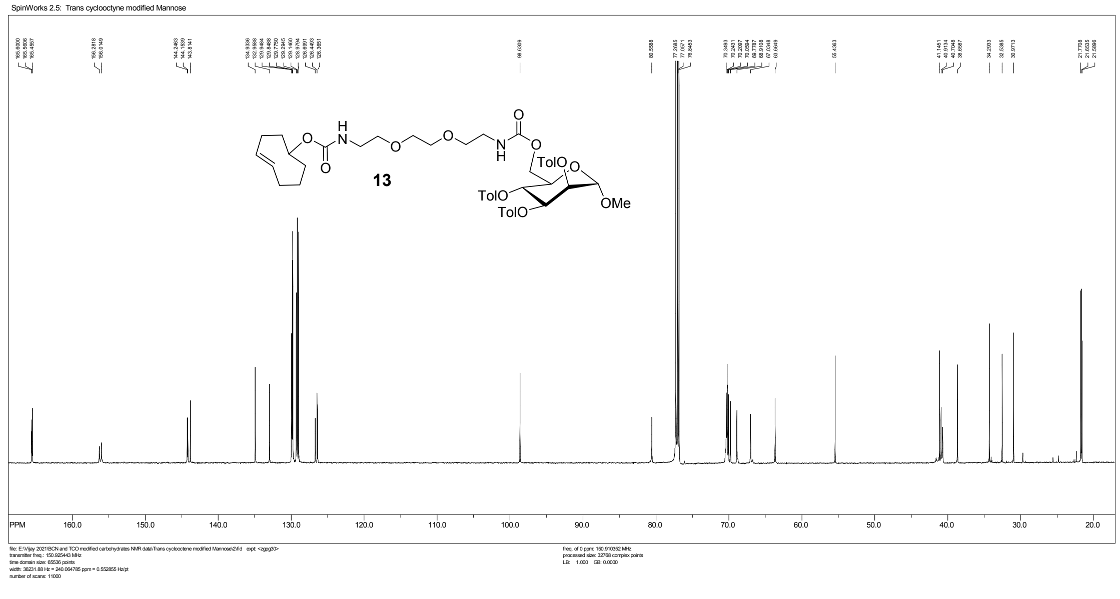

Figure S11. ^1H and ^{13}C NMR (600/150 MHz, $CDCl_3$) spectra of **13**.

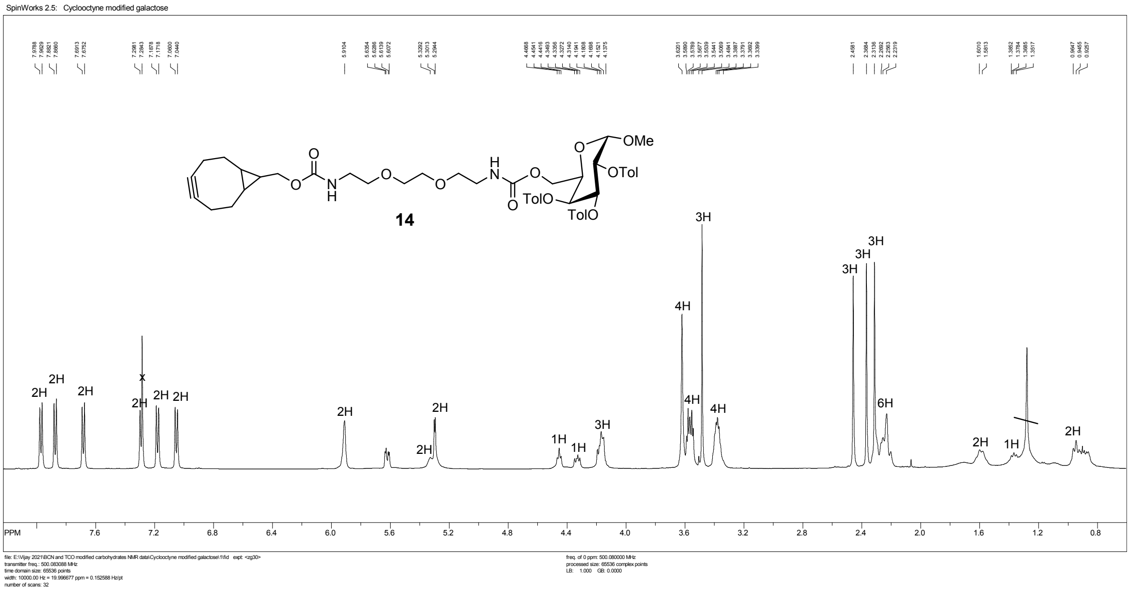

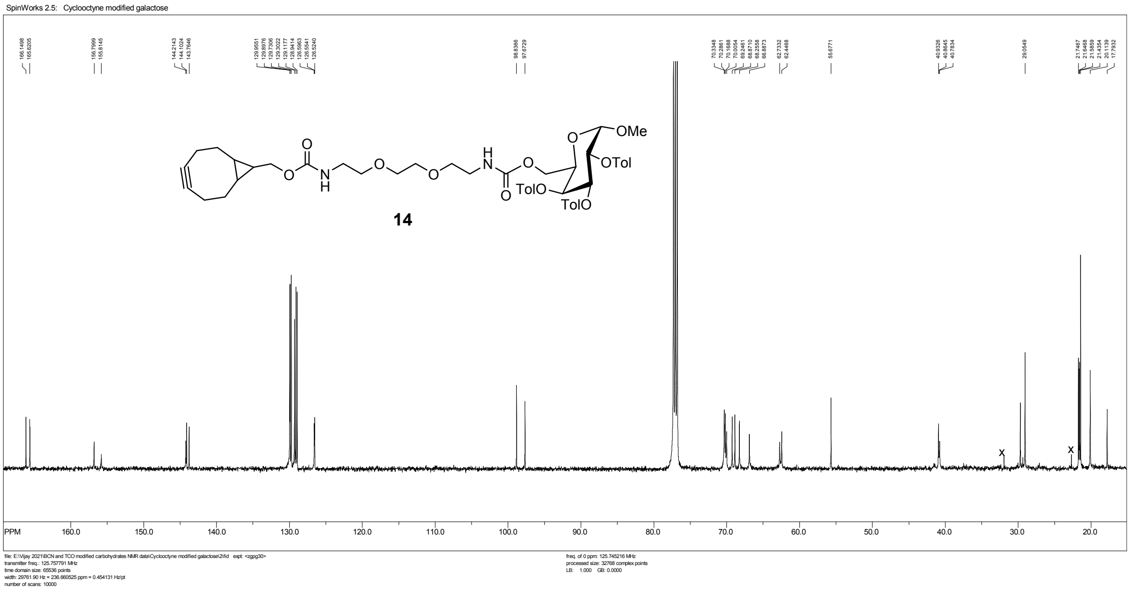

Figure S12. ^{1}H and ^{13}C NMR (500/125 MHz, $CDCl_3$) spectra of **14**.

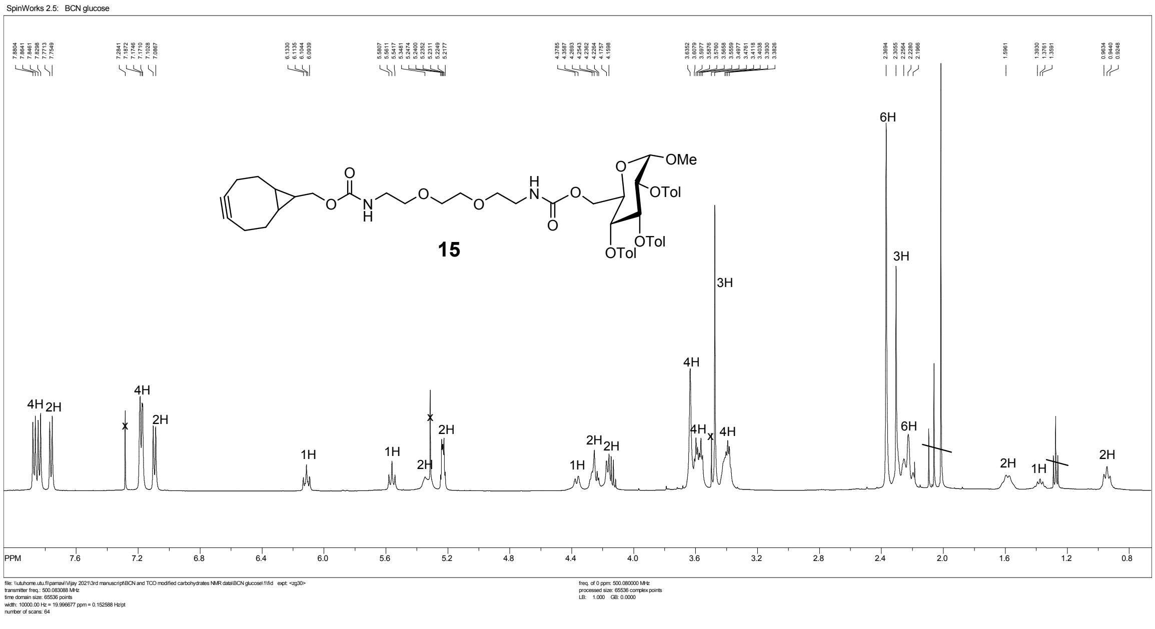

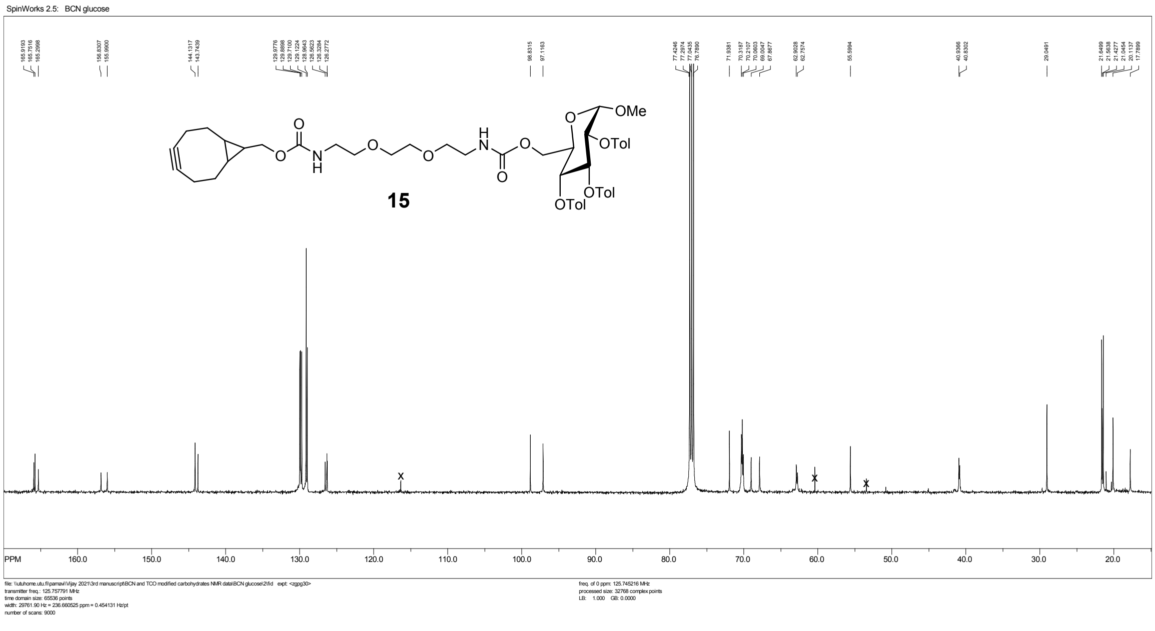

Figure S13. ^{1}H and ^{13}C NMR (500/125 MHz, $CDCl_3$) spectra of **15**.

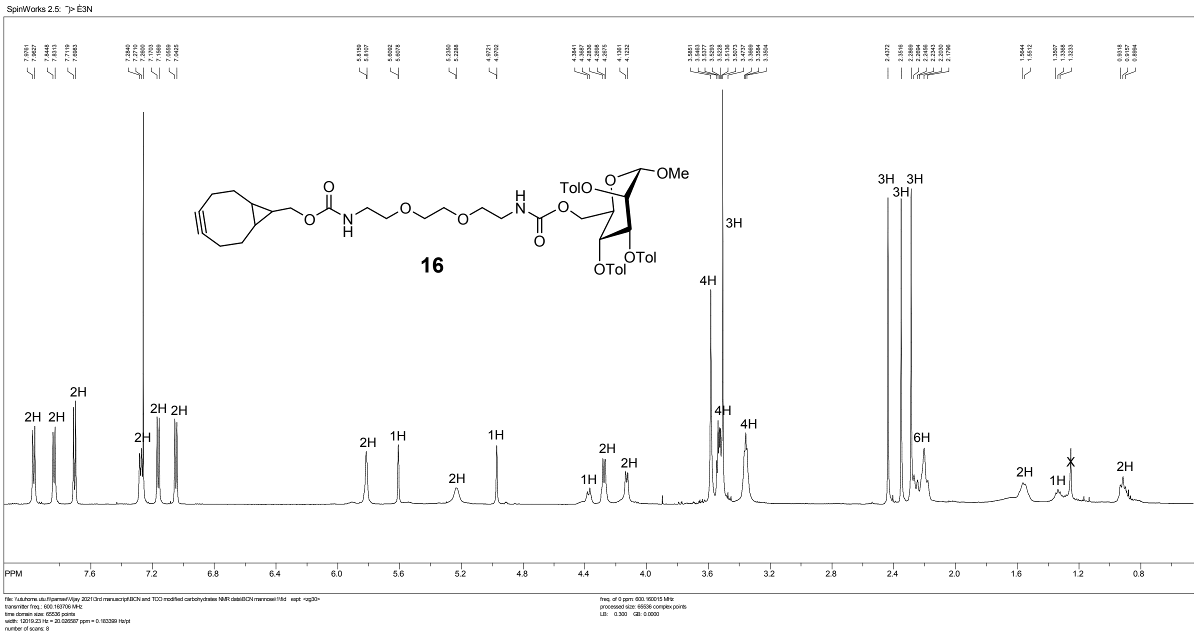

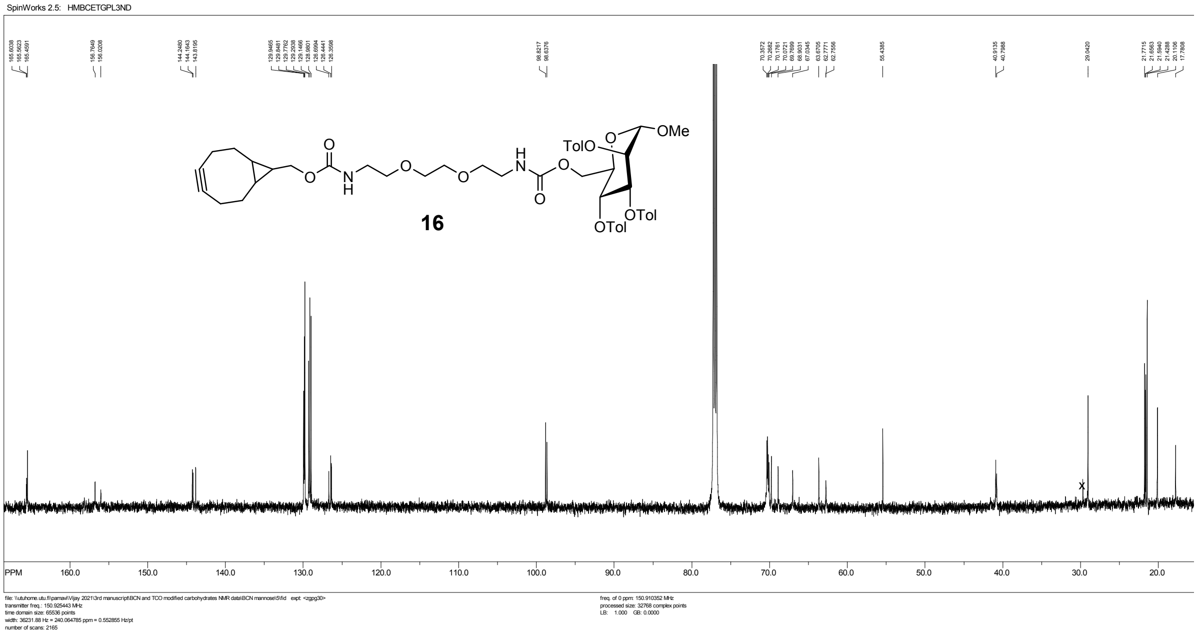

Figure S14. ^{1}H and ^{13}C NMR (500/125 MHz, $CDCl_3$) spectra of **16**.

Synthesis TCO- and BCN-modified oligonucleotides (TCO-ON1 and BCN-ON2) and BCN-modified peptide.

The 3'- and 5'-amino-modified oligonucleotides (**ON1** and **ON2** with aminohexyl spacer) were purchased from Metabion (https://www.metabion.com/). The amino-modified peptide (2-(2-(2-aminoethoxy)ethoxy)acetyl-Gly-Phe-Trp-Phe-Gly-NH_2) was synthesized by an automated peptide synthesizer (CEM, Liberty Blue) using the commercially available amino acid building blocks and Rink-amide ChemMatrix resin. Standard carbamate-coupling protocol was followed to obtain the desired activated oligonucleotide and peptide precursors: The amino modified oligonucleotides and the peptide (0.1 µmol) were dissolved in 100 µL of DMSO:H_2O (9:1). DIEA (diisopropyl ethyl amine, 0.2 µmol) and commercially available bicyclo[6.1.0]non-4-yn-9-ylmethyl or (E)-cyclooct-4-en-1-yl NHS carbonate (1.0 µmol) were added to the mixtures. The mixtures were gently shaken overnight at room temperature and introduced then to an RP-HPLC (an analytical column (C18, 250 × 4.6 mm, 5 µm), at a flow rate 1.0 mL min^{-1} , detection at λ = 260 nm and)(280 nm, a gradient elution from 0 to 100% MeCN in 50 mmol L^{-1} triethylammonium acetate over 30 minutes was used for each) to obtain **TCO-ON1**, **BCN-ON2** and **BCN-peptide** in 52%, 55% and 58% isolated yield, respectively. The yields were determined according to UV-absorbance at λ = 260 nm (**TCO-ON1** and **TCO-ON2**) and at λ = 280 nm (**BCN-peptide**)[1]. The authenticity of the products was verified by MS (ESI-TOF). **BCN peptide**: $[M+H]^+$: observed 934.4 (expected 934.1), **TCO-ON1**: observed 3071.7 (calculated from $[(M-3H)/3]^{3-}$, expected 3072.2), **BCN-ON2**: observed 3085.8 (calculated from $[(M-2H)/2]^{2-}$, expected 3086.2).

Scheme S3. Synthesis of BCN-peptide, TCO-ON1 and BCN-ON2 (RP HPLC profiles of crude product mixtures described).

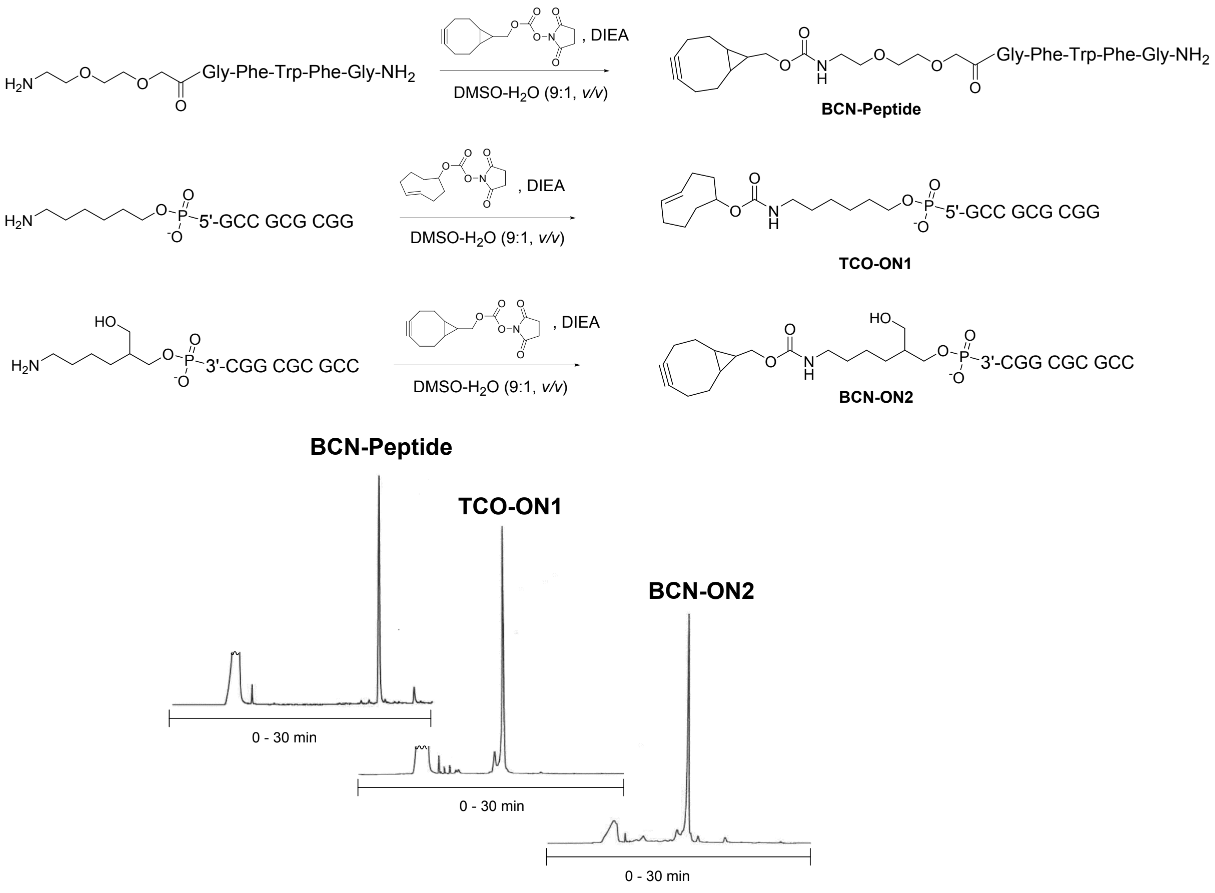

Scheme S4. RP HPLC analyses of the conjugates (see conditions in the general procedures)

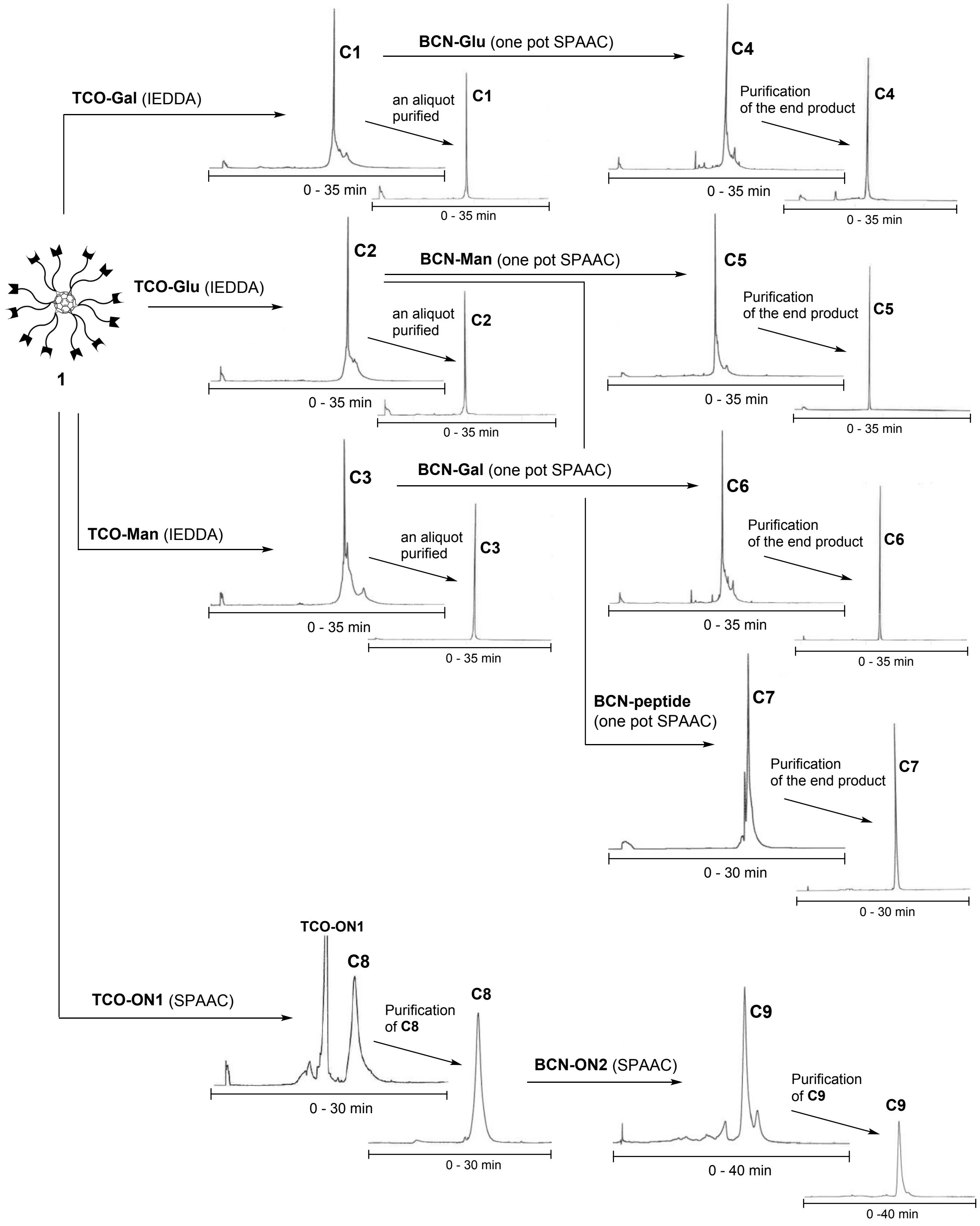

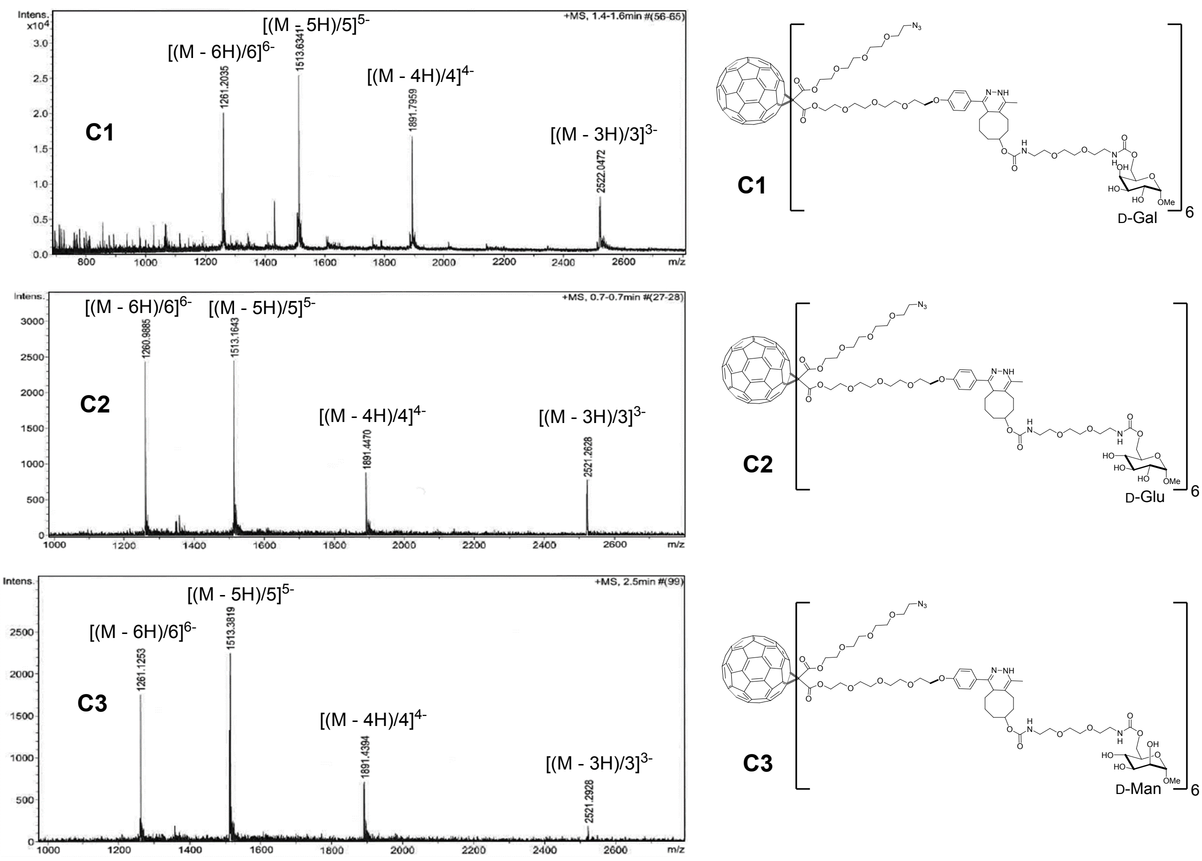

Figure S15. MS(ESI-TOF) spectra of C_{60}-glyco conjugates **C1-C3**. Observed molecular weights (calculated from $[(M-5H)/5]^{5-}$): 7573.2 (**C1**), 7570.9 (**C2**), 7571.9 (**C3**). Expected molecular weight: 7574.0.

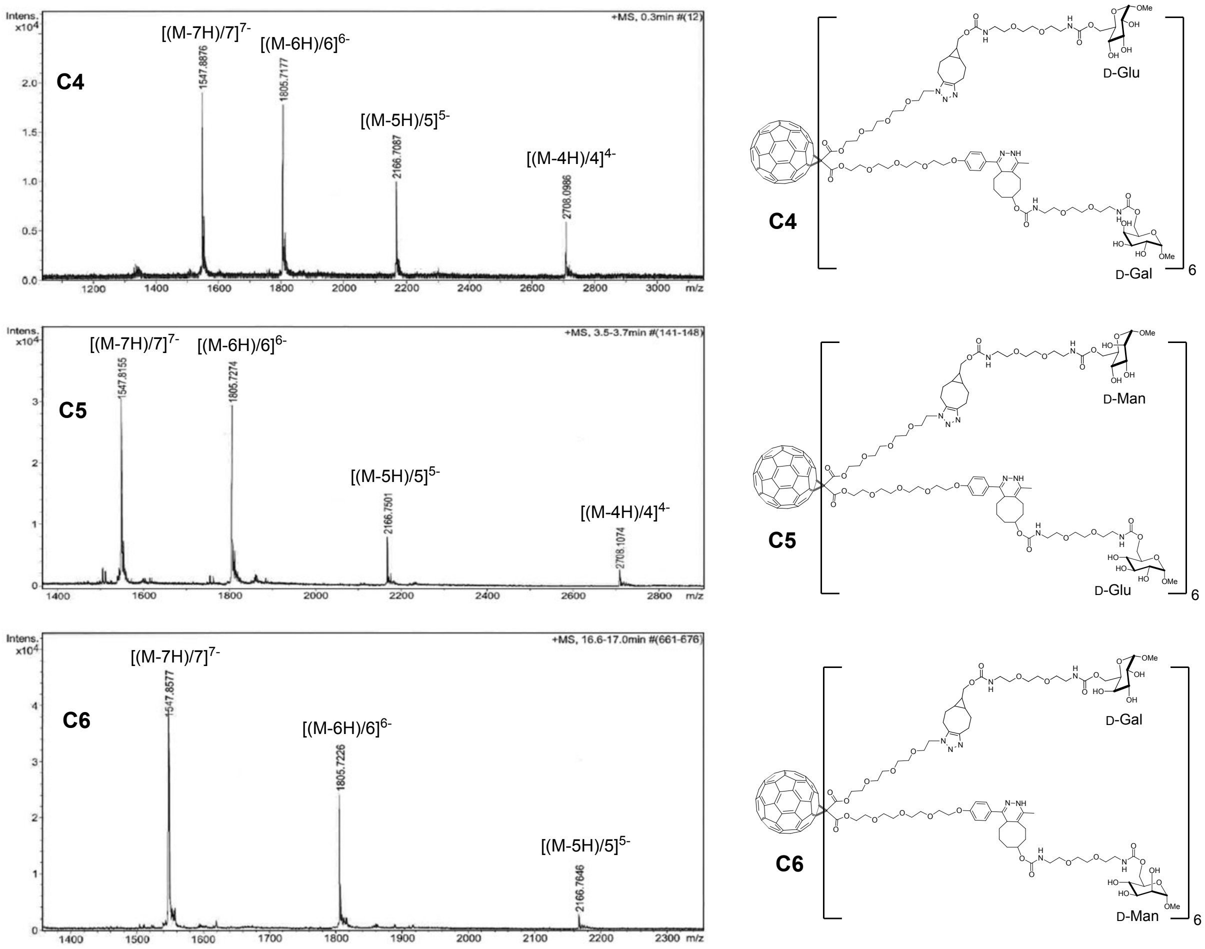

Figure S16. MS(ESI-TOF) spectra of C_{60}-glyco conjugates **C4-C6**. Observed molecular weights (calculated from $[(M-7H)/7]^{7-}$): 10842.3 (**C4**), 10841.8 (**C5**), 10842.1 (**C6**). Expected molecular weight: 10841.6.

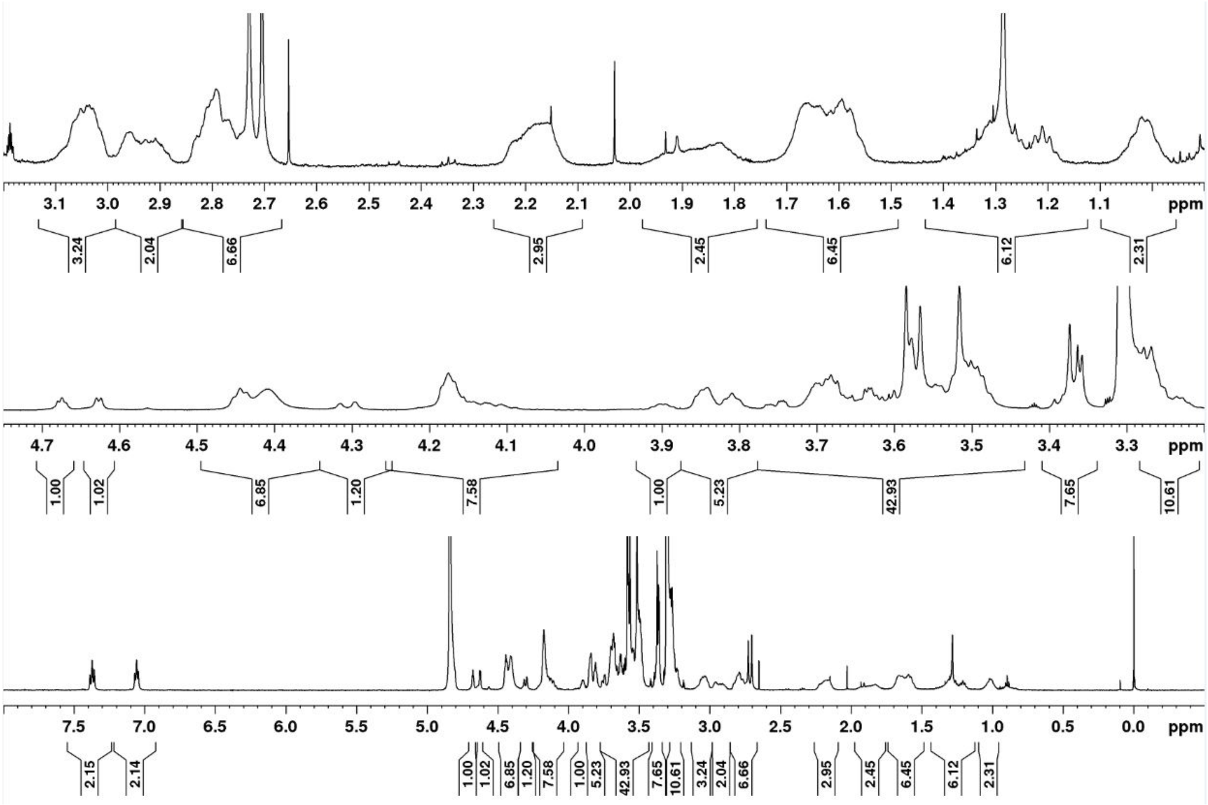

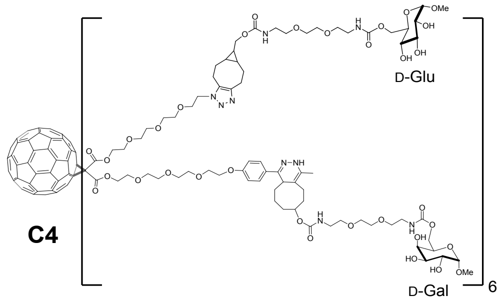

Figure S17. ^{1}H NMR (600MHz, d_6-DMSO)of **C4**.

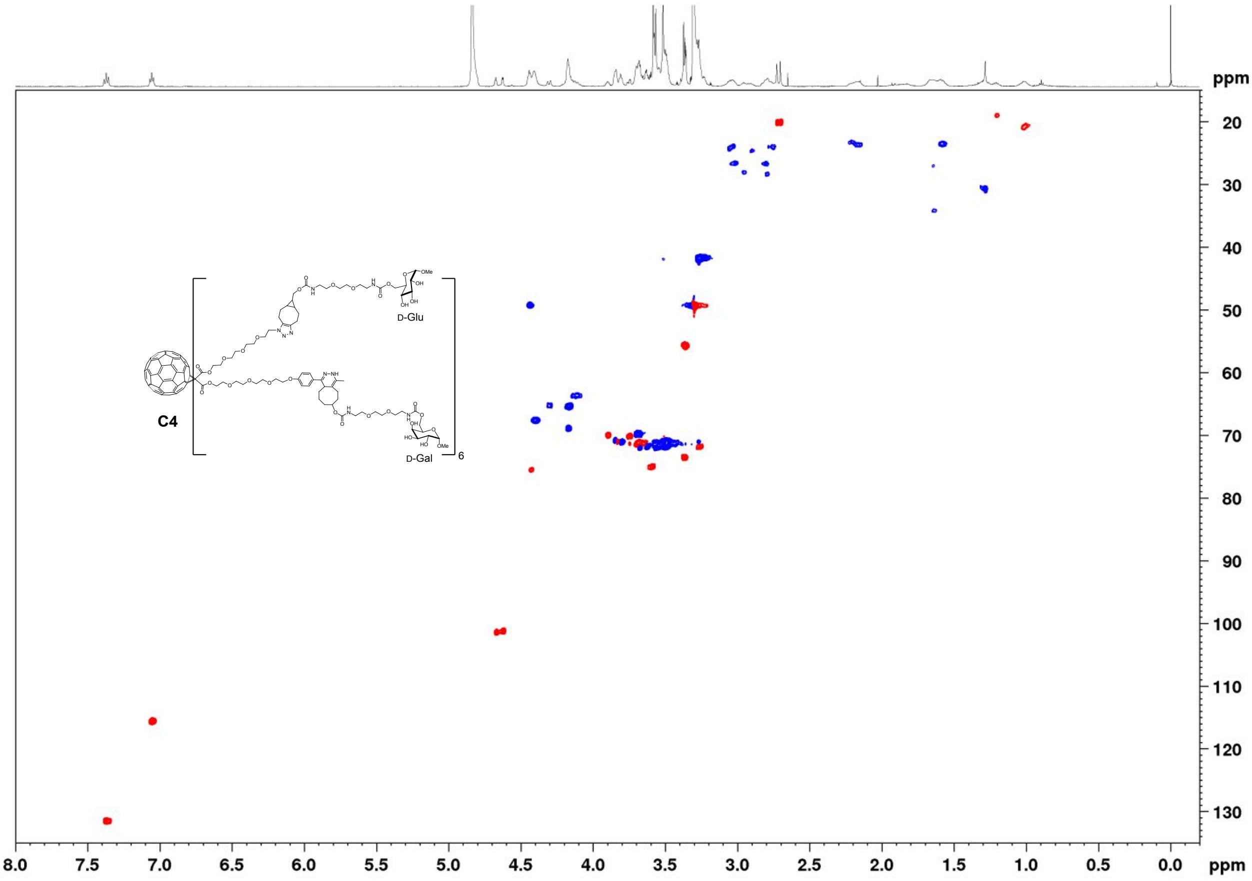

Figure S18. HSQC spectrum of **C4**.

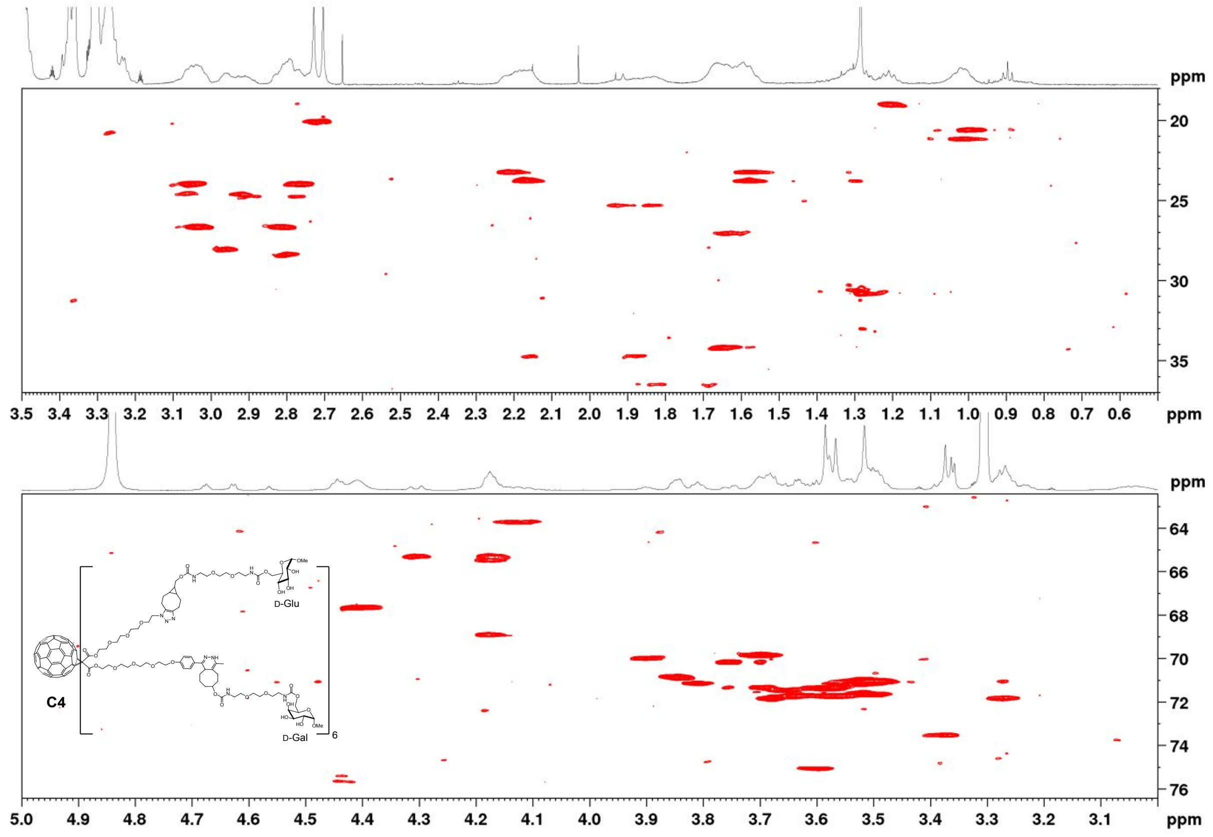

Figure S19. A band selective HSQC spectrum (SHSQC) of **C4**.

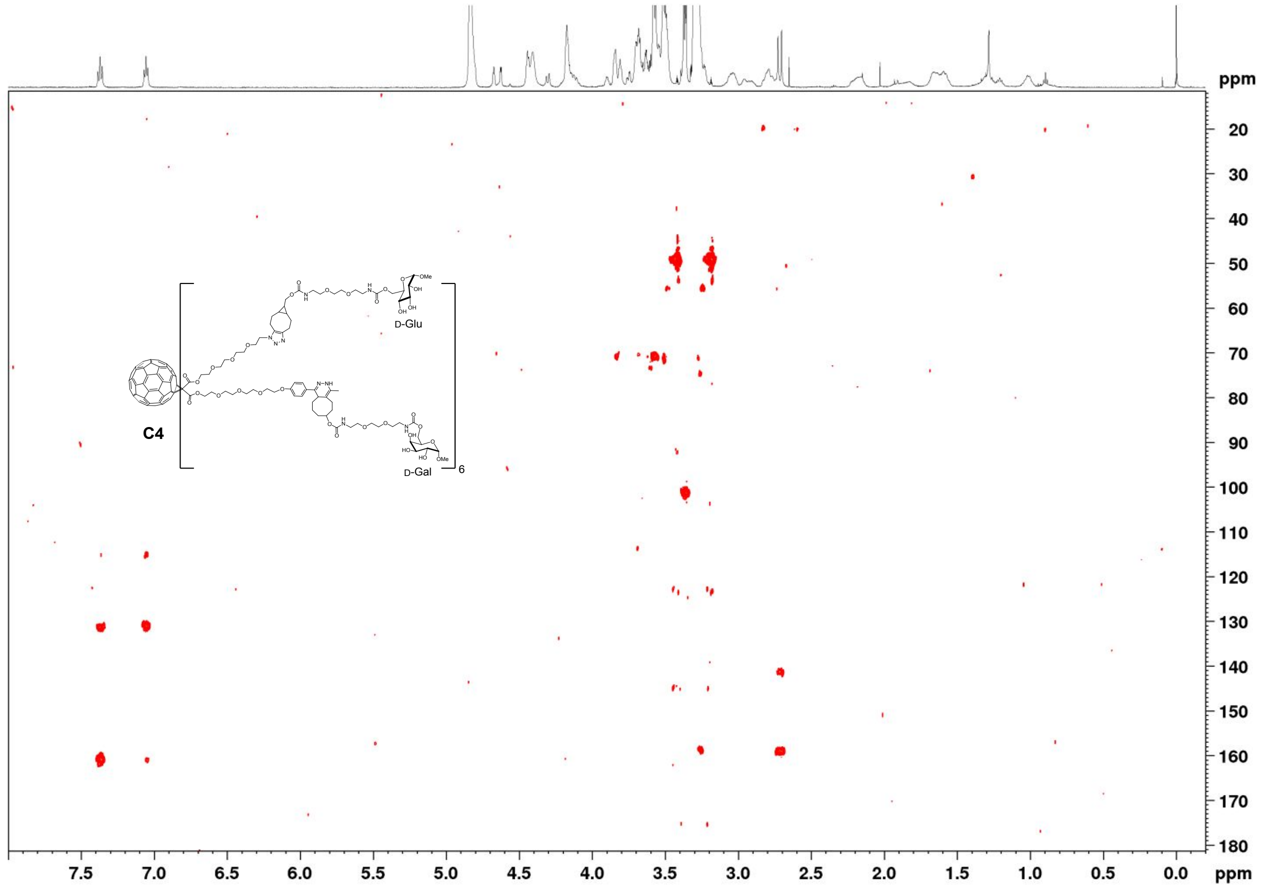

Figure S20. HMBC spectrum of **C4**.

Table S1. Assignments of ^{1}H and ^{13}C NMR shifts of **C4**

Position	δ_{1H} (ppm)	δ_{13C} (ppm)
-***C***OOCH$_2$CH$_2$O-		Not visible
-COO***CH$_2$***CH$_2$O-	4.40	67.7
-COOCH$_2$***CH$_2$***O-	3.69	69.8
-O***CH$_2$CH$_2$***O-	3.67, 3.63, 3.58, 3.57, 3.55, 3.51	71.8, 71.72, 71.73, 71.64, 71.35, 71.33
Ph-O***CH$_2$***CH$_2$O-	4.17	68.9
Ph-OCH$_2$***CH$_2$***O-	3.85	70.8
O-Ph-*i*		160.9
O-Ph-*o*	7.06	115.7
O-Ph-*m*	7.37	131.6
O-Ph-*p*		131.1
Ph-***C***-N		162.1
Ph-C=***C***		Not visible
Me-C=***C***		141.4
Me-***C***-N		159.2
Me	2.73, 2.70	20.12, 20.07
Pyr-***CH$_2$***CH$_2$CHOCH$_2$CH$_2$CH$_2$-	3.07, 2.93; 2.90, 2.77	24.6; 24.8
Pyr-CH$_2$***CH$_2$***CHOCH$_2$CH$_2$CH$_2$-	2.16, 1.88; 1.83, 1.68	34.7; 36.5
Pyr-CH$_2$CH$_2$***CH***OCH$_2$CH$_2$CH$_2$-	4.43	75.4, 75.7
Pyr-CH$_2$CH$_2$CHO***CH$_2$***CH$_2$CH$_2$-	1.65	34.2
Pyr-CH$_2$CH$_2$CHOCH$_2$***CH$_2$***CH$_2$-	1.93, 1.84;1.64, 1.61	25.3; 27.1
Pyr-CH$_2$CH$_2$CHOCH$_2$CH$_2$***CH$_2$***-	2.96; 2.80	28.0; 28.4
O***C***ON		158.8
OCON-***CH$_2$***CH$_2$O-	3.26	41.7
OCON-CH$_2$***CH$_2$***O-	3.51	71.0
Gal-1	4.67	101.5
Gal-2	3.75	70.2
Gal-3	3.69	71.3
Gal-4	3.84	70.9
Gal-5	3.90	70.0
Gal-6	4.17	65.5
Triaz-***CH$_2$***CH$_2$O-	4.44	49.2
Triaz-CH$_2$***CH$_2$***O-	3.81	71.1
Triazole-C		Not visible
Tri-***CH$_2$***CH$_2$CHCHCHCH$_2$CH$_2$-	3.06, 2.77	24.0
Tri-CH$_2$***CH$_2$***CHCHCHCH$_2$CH$_2$-	2.21, 1.58	23.2
Tri-CH$_2$CH$_2$***CH***CHCHCH$_2$CH$_2$-	1.00	20.6
Tri-CH$_2$CH$_2$CH***CH***CHCH$_2$CH$_2$-	1.21	19.0
Tri-CH$_2$CH$_2$CHCH***CH***CH$_2$CH$_2$-	1.01	21.2
Tri-CH$_2$CH$_2$CHCHCH***CH$_2$***CH$_2$-	2.17, 1.58	23.8
Tri-CH$_2$CH$_2$CHCHCHCH$_2$***CH$_2$***-	3.03, 2.81	26.7
-***CH$_2$***-OCON-	4.12	63.7
Glc-1	4.63	101.2
Glc-2	3.37	73.5
Glc-3	3.60	75.1
Glc-4	3.26	71.9
Glc-5	3.66	71.4
Glc-6	4.30, 4.17	65.3

NMR assignments of the arms. The fullerene core, and the closest quaternary carbon are not visible in HSQC or HMBC spectra due to being more than three bonds away from protons. Additionally some other signals are also not visible due to being far from protons, having unfavorable relaxation rates or being broadened by molecular motion or diastereoisomerism.

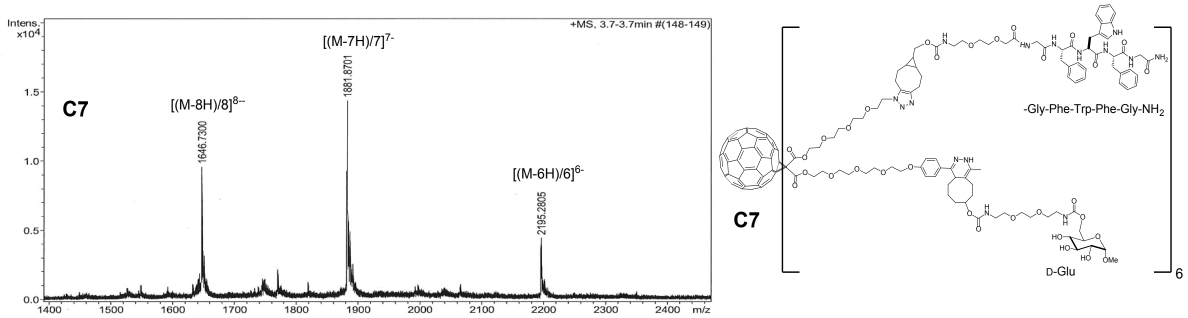

Figure S21. MS(ESI-TOF) spectrum of C_{60}-glyco/peptide conjugate **C7**. Observed molecular weight (calculated from $[(M-7H)/7]^{7-}$): 13180.1. Expected molecular weight: 13178.5.

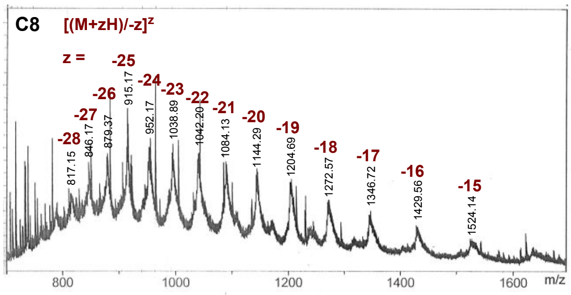

Expected multiply charged molecular ions

z	$[(M+zH)/-z]^z$
15:	1524.56
16:	1429.21
17:	1345.08
18:	1270.30
19:	1203.39
20:	1143.17
21:	1088.68
22:	1039.15
23:	952.47
24:	914.33
25:	879.13
26:	846.53
27:	816.26

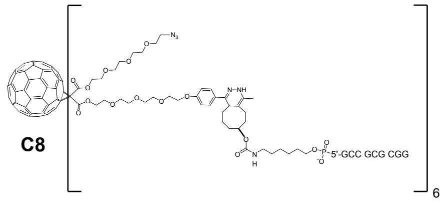

Figure S22. MS(ESI-TOF) spectrum of C_{60}-oligonucleotide conjugate **C8**. Observed molecular weight (calculated from $[(M-15H)/15]^{15-}$): 22877.2. Expected molecular weight: 22883.5.

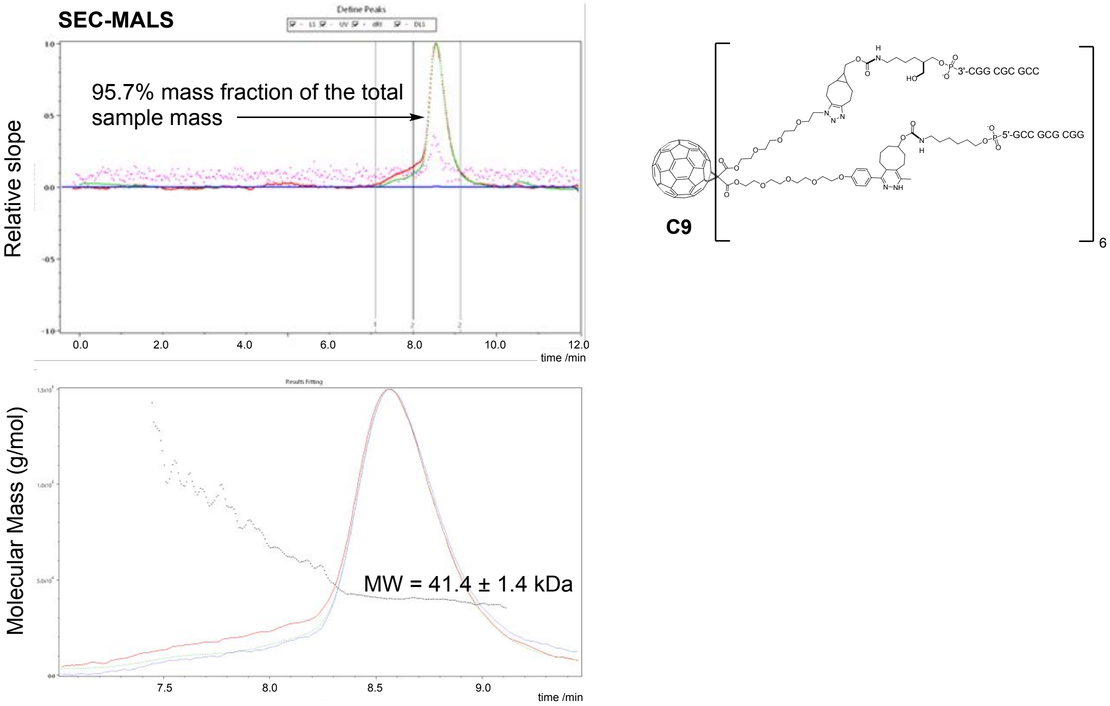

Figure S23. SEC-MALS (size exclusion chromatography equipped with multiple angle light scattering detector) analysis of **C9**. For the experimental details, see general procedures. Expected molecular mass: 41400 (41.4 kDa).

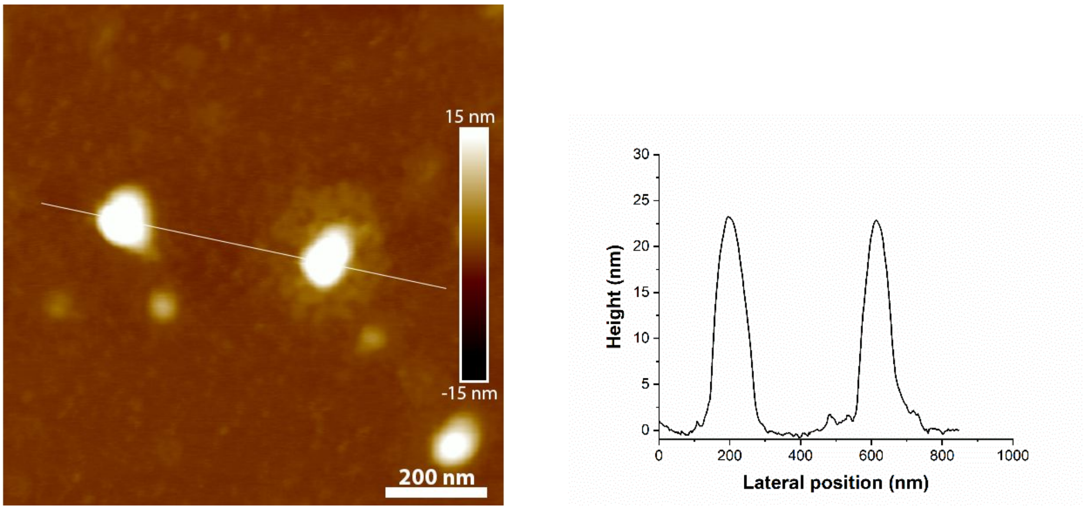

Figure S24. AFM height image of C9 on polyethyleneimine (PEI) coated mica in water, and a cross-section profile corresponding to the white line in the AFM image. In addition to the particles of 10 nm height, larger particles (ca 25 nm height) were observed on PEI-coated mica. What makes these data interesting is the controlled size of the aggregates, which may indicate self-assembled structures.

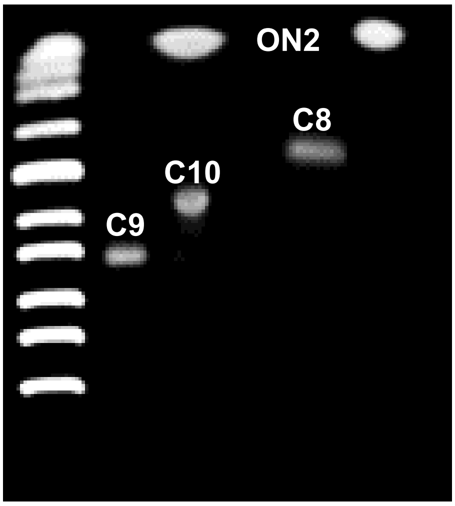

Figure S25. PAGE electrophoregram of C8, C9, C10 and ON2. An excess of **ON2** is used to form **C10** to show that the band of **C10** in the electrophoregram (cf. Figure 1) really is the end point. The different mobility between **C8** and **C10** is a surprising behavior as the only difference in these structures is the covalent link between **ON2** and the C_{60} core (**1**).

References

1) Delhoch, H. *Biochemistry* **1967**, *6*, 1948-1954.
2) Virta, P., Karskela, M., Lönnberg, H. *J. Org. Chem.* **2006**, *3*, 1989-1999.
